# Reduced R-loop abundance at proinflammatory loci: a shared epigenetic mechanism in inflammatory and metabolic diseases

**DOI:** 10.3389/fcvm.2026.1782258

**Published:** 2026-08-21

**Authors:** William Y. Yang, Fatma Saaoud, Keman Xu, Mohammed Ben Issa, Yifan Lu, Ying Shao, Baosheng Han, Xianwei Wang, Xiaohua Jiang, Sheng Wu, Laisel Martinez, Roberto I. Vazquez-Padron, Yinghui Zhong, Mingui Fu, Hong Wang, Xiaofeng Yang

**Affiliations:** 1 School of Communication and Information, Rutgers University, New Brunswick, NJ, United States; 2 Lemole Center for Integrated Lymphatics and Vascular Research, Department of Cardiovascular Sciences, Lewis Katz School of Medicine at Temple University, Philadelphia, PA, United States; 3 Centers of Metabolic Disease Research and Thrombosis Research, Department of Cardiovascular Sciences, Lewis Katz School of Medicine at Temple University, Philadelphia, PA, United States; 4 Department of Surgery, Leonard M. Miller School of Medicine, University of Miami, Miami, FL, United States; 5 School of Biomedical Engineering, Science and Health Systems, Drexel University, Philadelphia, PA, United States; 6 Department of Biomedical Science, School of Medicine, University of Missouri Kansas City, Kansas City, MO, United States

**Keywords:** hyperlipidemia, infection, inflammation, R-loop regulatory proteins, R-loops, ROS

## Abstract

**Introduction:**

R-loops, RNA-DNA hybrid structures with a displaced single-stranded DNA loop, are key regulators of transcriptional control, chromatin architecture, and genome stability and have emerging roles in inflammatory signaling. However, the relationship between R-loop abundance and strongly modulated inflammatory effector genes in metabolic inflammation and influenza virus infection remains underexplored.

**Methods:**

We performed a locus-centric integrative analysis combining robust differentially expressed genes (DEGs) from multiple inflammatory and infection-related murine and human transcriptomic disease models with experimentally validated multi-cell R-loop annotations from the reference atlas RLoopBase. Our correlation framework evaluated the directional relationship between R-loop abundance and inflammatory gene expression rather than assuming disease-sample-matched R-loop measurements. We further analyzed R-loop regulatory proteins, NRF2-associated R-loop regulators, and overlaps between R-loop regulators and CRISPRi-identified mitochondrial and cellular reactive oxygen species (ROS) regulators.

**Results:**

In angiotensin II-infused apolipoprotein E-deficient (ApoE−/−) mice, a model of abdominal aortic aneurysm (AAA), genomic regions encoding the top significantly upregulated genes exhibited significantly fewer R-loops than those encoding downregulated genes at days 14 and 28. Similarly, in atherosclerotic ApoE−/− mice fed a high-fat diet for 32 and 78 weeks, upregulated genes were associated with fewer R-loops than downregulated genes. Reduced R-loop abundance was also observed in genomic regions encoding the top significantly upregulated genes in liver tissues from patients with non-alcoholic steatohepatitis (NASH), as well as in monosodium urate (MSU)-stimulated lymphatic endothelial cells (LECs) and influenza virus-infected human umbilical vein endothelial cells (HUVECs). R-loop regulatory proteins upregulated during metabolic inflammation were enriched in immune and inflammatory pathways. NRF2 was identified as a regulator of 27 R-loop regulatory proteins, including 10 positively and 17 negatively regulated proteins. Furthermore, 54 R-loop regulatory proteins overlapped with CRISPRi-identified mitochondrial and cellular ROS regulators, suggesting potential reciprocal regulation between R-loop homeostasis and ROS signaling. Disease-associated changes in pro-ROS and anti-ROS R-loop regulatory proteins further linked R-loop regulation to inflammatory and oxidative stress pathways.

**Discussion:**

These findings identify reduced R-loop abundance at genomic regions encoding strongly upregulated inflammatory genes as a shared feature across multiple models of metabolic inflammation and influenza virus infection. The results further suggest that immune-associated R-loop regulatory proteins and the NRF2–ROS axis may contribute to R-loop remodeling during inflammatory disease. This integrative framework provides new insight into the potential role of R-loops and ROS-sensitive R-loop regulators in inflammatory and metabolic diseases and identifies candidate pathways for future mechanistic investigation and therapeutic targeting.

## 1 Introduction

**Figure 1:**
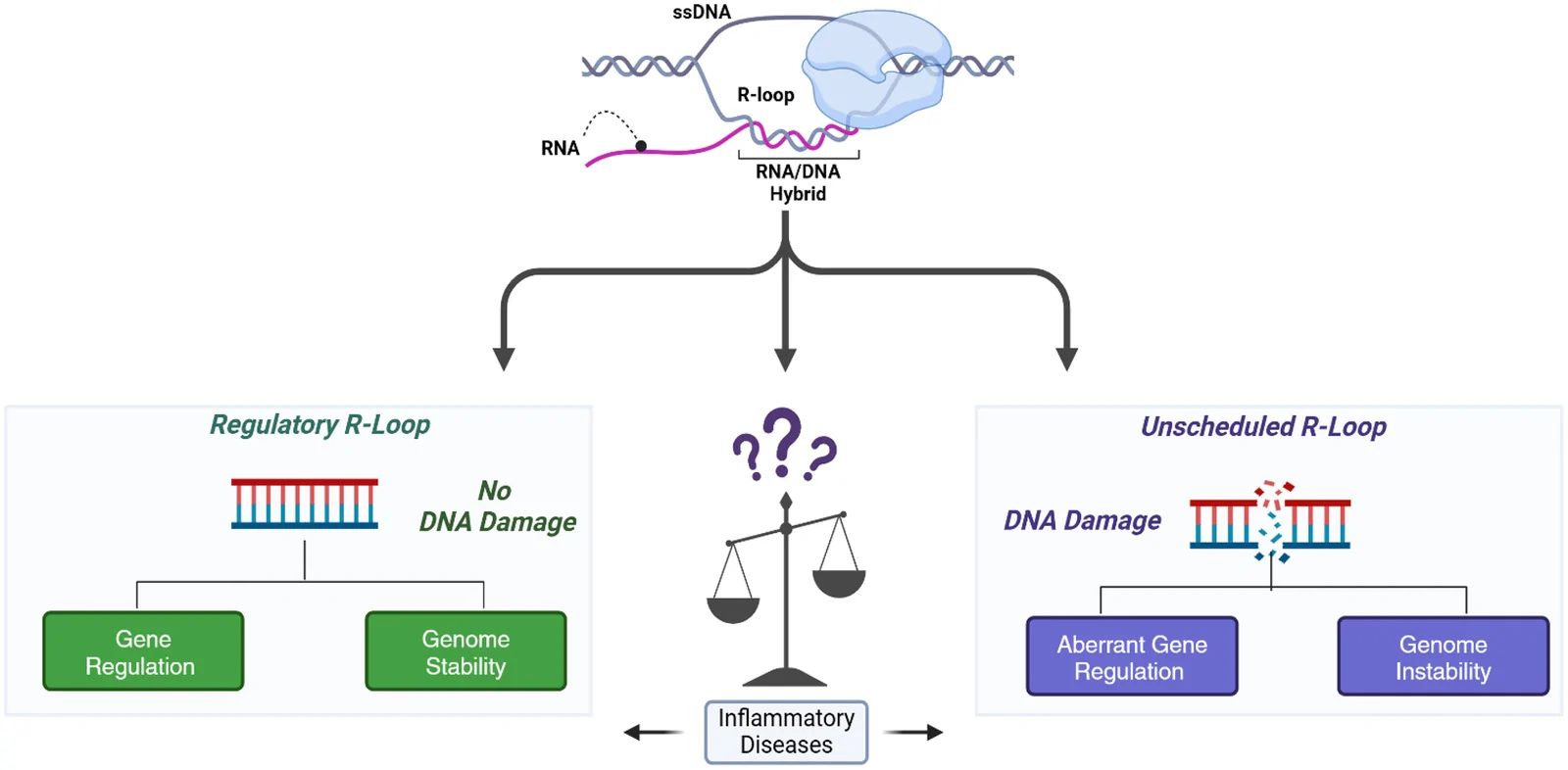
Conceptual framework illustrating the dual roles of R-loops in genome instability and gene regulation, and their context-specific patterns in inflammatory diseases. R-loops are three-stranded nucleic acid structures consisting of an RNA/DNA hybrid and a displaced single-stranded DNA strand. These structures can form either aberrantly (unscheduled) or in regulated, physiological manner. Unscheduled R-loops are associated with genome instability, DNA damage, and contribute to the pathogenesis of inflammation-related diseases. In contrast, regulatory R-loops play essential roles in modulating gene expression and chromatin architecture under normal cellular conditions. Despite their significance, the precise functions and dynamics of R-loops in the initiation and progression of inflammatory diseases remain poorly understood, representing a key gap in current knowledge.

R-loops are three-stranded nucleic acid structures composed of an RNA-DNA hybrid and a displaced single-stranded non-template DNA strand. These structures are formed co-transcriptionally when the nascent RNA hybridizes with the DNA template strand (1) (Figure 1). In mammalian genomes, R-loops occupy approximately 3%–5% of the genome (2). R-loops can exert both beneficial and deleterious effects on biological processes and are therefore classified into two categories, unscheduled (or unprogrammed) R-loops and regulatory R-loops. Unscheduled R-loops arise under pathological conditions and are often associated with genome instability (3, 4). These unscheduled R-loops may serve as molecular triggers or amplifiers of intra-nuclear danger signals (5, 6).

R-loop homeostasis is controlled by a conserved class of R-loop regulatory proteins, which actively govern hybrid formation, stabilization, or removal. These include: (i) RNA:DNA helicases such as Senataxin, which resolve R-loops and prevent transcription-replication conflicts (7); (ii) RNase H enzymes such as RNase H2, which specifically degrade RNA within hybrids (8); (iii) Topoisomerases including TOP1, which limit supercoil-driven hybrid accumulation (9); and (iv) RNA-binding and splicing factors like SRSF1 that suppress unscheduled hybrids by coordinating RNA maturation (5, 10). Additional regulatory readers and architectural proteins, including BRCA1 and Coilin, anchor hybrid control to nuclear sub-domains and disease-relevant transcriptional programs (6). Under oxidative or immune stress, these proteins are themselves dynamically transcriptionally reprogrammed, linking R-loops regulation with ROS-sensitive gene networks, immune licensing, and epigenomic remodeling. As shown in Table 1, R-loop accumulation acts as a key mediator of genomic stress and nuclear inflammation and injuries (11). In contrast to detrimental effects of unscheduled R-loops, regulatory R-loops function as important components of gene regulation, facilitating processes such as transcription termination, epigenetic modulation, DNA repair, and telomere stability (Figure 1).

**Table 1:** R-Loop accumulation, genome instability, and disease: summary of factors, pathologies, and functional impacts.

Genome stabilizers and R-loop suppressor	R-loops pathology	R-loops function	PMID
NABP2 (encoding SSB1) KO or cancer-associated mutations into its intrinsically disordered region	Accumulation of R-loops	R-loops affect genome-stability regulator sensor of single-stranded DNA (SOSS)–the transcription regulator Integrator-PP2A (INTAC) localization	37557913
Acute liver damage in the setting of targeted Splicing factor SRSF1 deletion	Excessive formation of deleterious RNA-DNA hybrids (R-loops)	Excessive R-loops induce DNA damage	36759613
Senataxin (SETX) is an RNA/DNA helicase which catalyzes the unwinding of RNA:DNA hybrid portion of the R-loops, promoting thus their resolution	Gain or loss of function SETX mutations underlie the pathogenesis of two distinct neurological disorders AOA2 (ataxia with oculomotor apraxia—type 2) and ALS4 (juvenile Amyotrophic Lateral Sclerosis), respectively	R-loops formation can impact numerous biological processes,such as antibodies diversification in B cells, DNA damage response and gene expression at multiple levels, including regulation of the chromatin architecture at the promoter region, transcription elongation, and termination	37147318
HSF4 and COIL can form a complex under UV irradiation, and the preference for binding target genes changed	The presence of a large number of R-loops in cells under UV irradiation and the ability of COIL to recognize R-loops.	N6-(2-hydroxyethyl)-adenosine can competitively bind COIL and inhibit the binding of COIL to the R-loop. Thus, the activation of downstream inflammation-related genes and inflammatory skin injury was inhibited	37461263
Disruption of nuclear TDP-43 caused excessive R-loop accumulation	Excessive R-loop accumulation, which triggered DNA damage and genome instability and thereby induced PARP1 hyperactivation, leading to subsequent NAD + depletion and ATP loss, consequently activating mitochondrion-dependent necroptosis in intestinal epithelial cells.	Leading to subsequent NAD + depletion and ATP loss, consequently activating mitochondrion-dependent necroptosis in intestinal epithelial cells.	38157451
Cancers	ATM, ATR, cGAS/STING, and noncanonical pathways are the main pathways that involve in the regulatory network of R-loops in cancer. Regulation of R-loops induces kinds of drug resistance in various cancers, suggesting that targeting R-loops may be a promising way to overcome treatment resistance.	R-loops significantly influence cancer development, progression and treatment efficiency by regulating key genes, such as PARPs, BRCA1/2, sex hormone receptors, DHX9, and TOP1. Cancer biology can be modulated by R-loops-regulated phenotypes, including RNA methylation, DNA and histone methylation, oxidative stress, immune and inflammation regulation, and senescence. The role of R-loops in tumorigenesis remains controversial, and senescence may be a crucial research direction to unravel the mechanism of R-loop-induced tumorigenesis.	39613219

R-loops can modulate chromatin architecture by influencing histone post-translational modification and may be recognized by specific RNA/DNA hybrid-binding “reader” proteins. A recent study demonstrated that endosomal accumulation of N^4^-acetylcytidine (ac^4^C)-unmodified RNAs—likely originating from R-loops—activates inflammatory responses (12). These findings highlight the dual roles of R-loops in both maintaining cellular homeostasis and driving inflammation. Thus, the formation, localization, and removal of R-loops must be tightly regulated (13). Since this field is still in its infancy, key questions remain unresolved—particularly regarding how metabolic stress, inflammatory disease risk factors, and pathogen-/danger-associated molecular patterns (PAMPs)/(DAMPs) (14) recognized by receptors on plasma membrane and intracellular organelles influence regulatory R-loop dynamics in various pathologies. However, how DAMP signaling from extra-nuclear compartments (e.g., plasma membrane, cytosol and intracellular organelles) influences regulatory R-loops in inflammation and diseases is still unknown.

Regardless of significant progress, several important questions remain to be addressed including: *first*, whether decreased R-loops are associated with upregulation of proinflammatory genes in metabolic cardiovascular diseases, lymphatic vascular diseases, liver diseases, gouty arthritic, and viral infections? Whether increase or decrease number of R-loops in metabolic cardiovascular diseases, and viral infections associated with the expression of certain R-loops regulator? Whether influenza virus infection and monosodium urate (MSU), a known gouty arthritis inducing risk factor and NOD-, LRR- and pyrin domain-containing protein 3 (NLRP3) inflammasome activator (15, 16), affect R-loop regulator expression? Whether NRF2 (NFE2 Like BZIP Transcription Factor 2), an antioxidative transcription factor (TF) (17), modulates R-loops regulator expression?.

**Figure 2:**
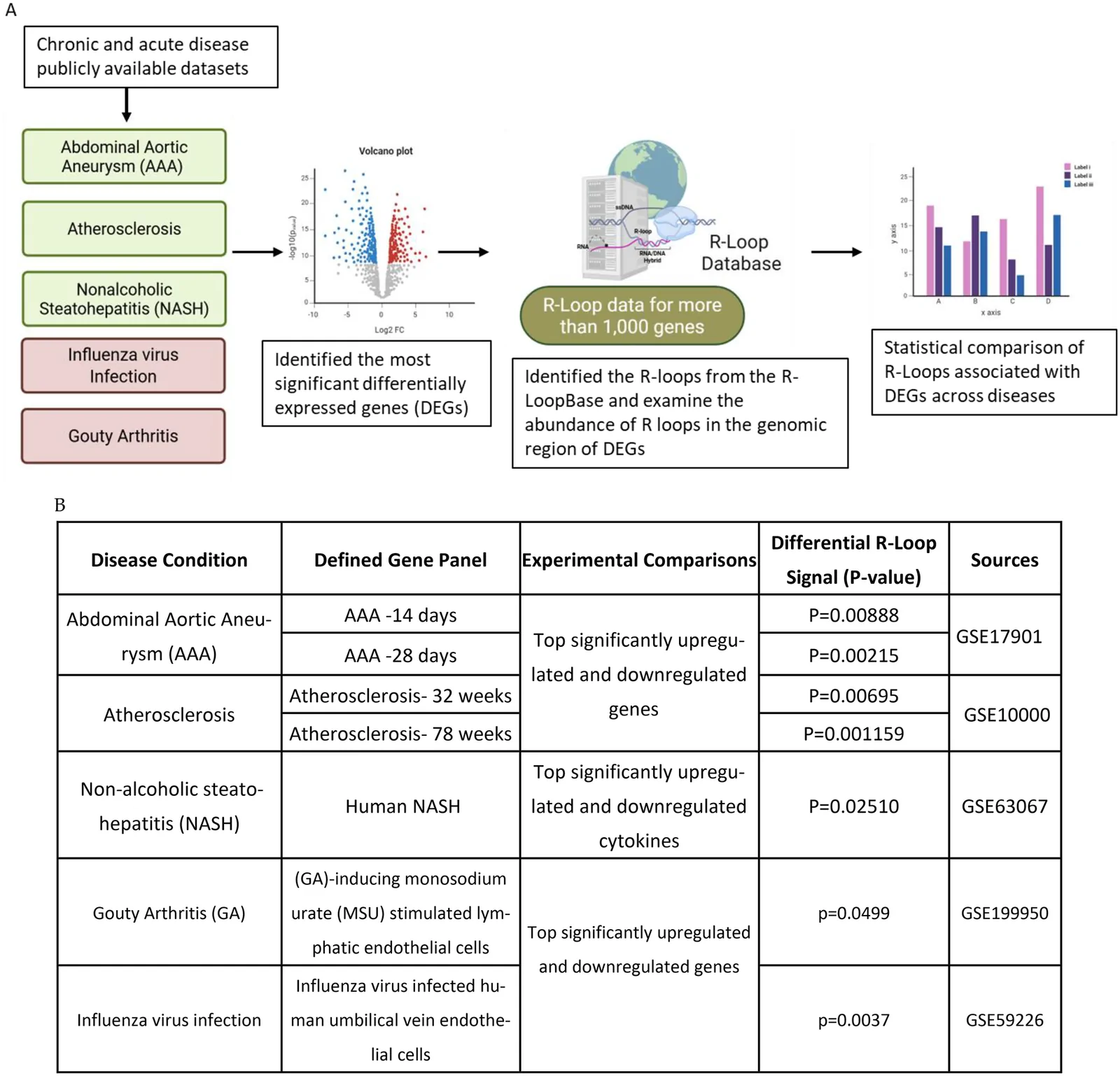
Study design and transcriptomic datasets analyzed. **(A)** Workflow overview of the analytical pipeline. Publicly available transcriptomic datasets were retrieved from the NIH NCBI Gene Expression Omnibus (GEO), encompassing both chronic and acute disease models, including abdominal aortic aneurysm (AAA), atherosclerosis, nonalcoholic steatohepatitis (NASH), influenza virus infection, and gouty arthritis. Differentially expressed genes (DEGs) were identified within each dataset using thresholds of log₂ fold change (FC) >1 and *P* < 0.05. DEG were then cross-referenced with the R-loopBase database to extract corresponding R-loop numbers in the genomic expression region of those DEGs. Comparative statistical analyses (two-tailed *t*-tests) were performed between upregulated and downregulated gene groups across disease models to identify R-loop signatures. **(B)** Overview of the RNA-Seq datasets included in the study. The datasets span diverse pathological contexts, including cardiovascular disease, metabolic liver disease, autoimmune inflammation, and acute viral infection. Among the top significantly upregulated and downregulated genes in each dataset, those exhibiting significant alterations in R-loop numbers were identified and evaluated.

Using publicly available sequencing data (18, 19), we performed integrative analysis of disease-modulated genes and their associated R-loop structures (Figure 2). Our results have suggested that R-loop dysregulation may contribute to the pathogenesis of chronic inflammatory diseases including atherosclerosis, abdominal aortic aneurysm (AAA), NASH, gouty arthritis, and acute inflammation such as influenza virus infection. Mechanistically, these effects may be mediated by ROS-regulated TF-modulated R-loop regulator pathways, providing a novel link between oxidative stress, inflammation, and genome instability. These findings offer important insights into the role of R-loops in chronic and acute inflammations and identify potential therapeutic targets for treating cardiovascular, metabolic and immune-mediated diseases, transplantation-related complications, aging, and cancer.

## 2 Results

### 2.1 R-loop numbers are significantly decreased in the genomic regions of upregulated genes during AAA progression

**Figure 3:**
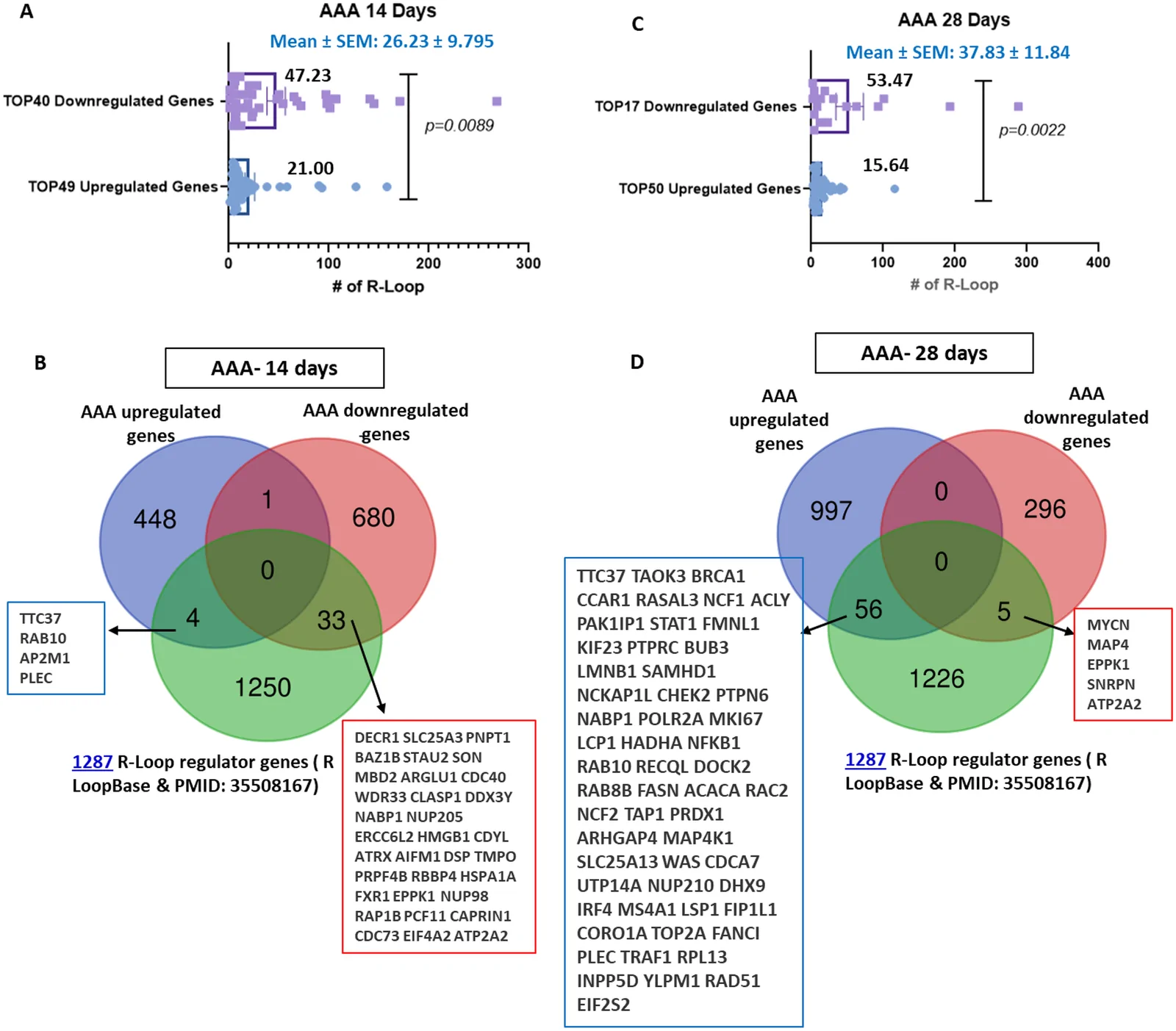
Increased R-loop numbers in downregulated genes during angiotensin II-induced abdominal aortic aneurysm (AAA). **(A)** In the 14-day AngII-infused AAA model, the top 40 significantly downregulated genes exhibited significantly higher R-loop numbers compared with the top 49 upregulated genes (*p* = 0.0089). **(B)** Venn diagram illustrates the overlap between R-loop regulatory proteins and differentially expressed genes at day 14. Four upregulated genes and 33 downregulated genes overlapped with known R-loop regulatory factors, highlighting R-loop–related pathways enriched among downregulated targets. **(C)** In the 28-day AngII-infused AAA model, the top 17 significantly downregulated genes displayed significantly higher R-loop numbers compared with the top 50 upregulated genes (*p* = 0.0022). **(D)** Venn diagram shows overlap between R-loop regulatory proteins and differentially expressed genes at day 28. Fifty-six upregulated genes and five downregulated genes overlapped with known R-loop regulatory factors, revealing distinct pathway involvement at this later stage. Transcriptomic data for AngII-induced AAA were obtained from a publicly available dataset (4). R-loop regulatory proteins were compiled from R-loopBase and experimentally validated R-loop studies (23).

To investigate the involvement of R-loops in the pathogenesis of AAA, we analyzed R-loop numbers in the genomic regions encoding differentially expressed genes following angiotensin II (Ang-II) infusion in apolipoprotein E-deficient (ApoE^−^/^−^) mouse aorta, a well-established murine model of AAA (20) after 14 and 28 days. Using transcriptomic data (RNA-Seq) from the NIH-NCBI GEO dataset GSE17901 (21), we collected the top significantly upregulated and top significantly downregulated genes at 14 and 28 days after Ang-II infusion. We hypothesized that R-loops may be altered in the genomic regions encoding these differentially expressed genes in AAA aortas. To test this, we queried the R-loopBase database (https://rloopbase.nju.edu.cn/) (19, 22), and analyzed the R-loop numbers in the genomic regions of top significantly upregulated genes and top significantly downregulated genes identified in mouse aortas from Ang-II infusion-induced AAA in ApoE^−^/^−^ mice, 14 days and 28 days after Ang II infusion. At 14 days post-Ang-II infusion, we found that the R-loop numbers in the genomic regions of the top 49 upregulated genes were significantly lower than those of the top 40 downregulated genes (*p* < 0.0089) (Figure 3A). At 28 days, the R-loop numbers in the genomic regions encoding for the top 50 upregulated genes were significantly reduced compared to the top 17 downregulated genes (*p* < 0.0022) (Figure 3C).

Our previous studies (4) identified significant upregulation of proinflammatory cytokines, chemokines, innate immune molecules, and surface markers (e.g., plasma membrane proteins and clusters of differentiation (CD) markers) in these same transcriptomic datasets. The current findings suggest a negative correlation between R-loop numbers and inflammatory gene expression, implying that decreased R-loop formation in proinflammatory gene regions may be associated with their transcriptional activation during AAA development.

We next hypothesized that the expression of R-loop regulatory proteins is also modulated during AAA pathogenesis. To test this hypothesis, we collected R-loop regulatory proteins including a list of 52 experimentally validated R-loop regulatory proteins from a recent review in Molecular Cell (23), and an expanded list of 1,268 R-loop regulatory proteins identified in R-loopBase (19, 22) using R-loop-specific monoclonal antibody chromatin immunoprecipitation followed by DNA sequencing (CHIP-Seq) (https://rloopbase.nju.edu.cn/). Transcriptomic analysis of AAA aortas at 14- and 28-days post Ang-II infusion revealed the following patterns: At 14 days, four R-loop regulatory proteins were upregulated, including *RAB10* (1.14 log2FC, *p* = 0.04), *AP2M1* (1.14 log2FC, *p* = 0.002), *PLEC* (1.07 log2FC, *p* = 0.017), and *TTC37* (1.03 log2FC, *p* = 0.008), while 33 were downregulated. The top five downregulated R-loop regulatory proteins include *DECR1 (*−1.2 log2FC, *P* = 0.016), *PNPT1* (−1.08 log2FC, *p* = 0.02), *BAZ1B* (−1.06 log2FC, *p* = 0.0008), *SLC25A3* (−1.03 log2FC, *p* = 0.0005), and *STAU2* (−1.03 log2FC, *p* = 0.018) (Figure 3B).

On 28 days, 56 R-loop regulatory proteins were upregulated. The top five upregulated R-loop regulatory proteins include *CCAR1* (1.99 log2FC, *p* = 0.001), *RASAL3* (1.6 log2FC, *p* = 0.04), *TAOK3* (1.49 log2FC, *p* = 0.03), *TTC37* (1.4 log2FC, *P* = 0.03*),* and *BRCA1* (1.11 log2FC, *p* = 0.005), while five were downregulated, including *MAP4* (−2.75 log2FC, *p* = 0.03), *MYCN* (−2.15 log2FC and *P* = 0.01), SNRPN (−1.36 log2FC and *P* = 0.04), *EPPK1* (−1.3 log2FC and *P* = 0.02), *ATP2A2* (−1.05 log2FC and *P* = 0.003) (Figure 3D). Functional enrichment analysis revealed distinct signaling pathway profiles, the 33 downregulated R-loop regulatory proteins at 14 days were enriched in pathways, including mRNA metabolic process, cellular response to chemical stress, RNA localization, metabolism of RNA, and regulation of mRNA metabolic process. However, the 53 upregulated R-loop regulator y proteins at 28 days were associated with cell population proliferation, RAC1 GTPase cycle, microglia pathogen phagocytosis pathway, B-cell receptor signaling, and cortical cytoskeleton organization. These shifts in pathway enrichment indicate a transition from nuclear RNA processing dysfunction (day 14) to activation of immune and cytoskeletal responses (day 28). Strikingly, the ratio of upregulated to downregulated R-loop regulatory proteins followed a distinct up–down–up pattern that paralleled the previously reported biphasic expression pattern of proinflammatory molecules during AAA progression (4); AAA day 14 (4 upregulated/33 downregulated (ratio = 0.12)), and AAA day 28 (56 upregulated/5 downregulated (ratio = 11.2)).

These results suggest that R-loop regulators are dynamically expressed during AAA development, potentially contributing to the transcriptional reprogramming associated with inflammation and vascular remodeling. Furthermore, the signaling pathways of top modulated R-loop regulators started from downregulated mRNA processing and nuclear pore complex assembly at AAA 14 days and swing to upregulated extra-nuclear immune response pathways at AAA 28 days. Collectively, our findings demonstrate that R-loop numbers are significantly reduced in the genomic regions encoding genes upregulated during AAA progression at 14- and 28-days post-Ang-II infusion in ApoE^−^/^−^ mouse aortas. The expression of R-loop regulators is dynamically modulated, with distinct temporal patterns linked to inflammatory gene expression and signaling pathway transitions. These data provide the first evidence linking reduced R-loop formation and altered R-loop regulator expression to the transcriptional activation of proinflammatory genes in aortic aneurysm development.

### 2.2 The R-loop numbers are significantly decreased in the genomic regions of upregulated genes during atherosclerosis progression

**Figure 4:**
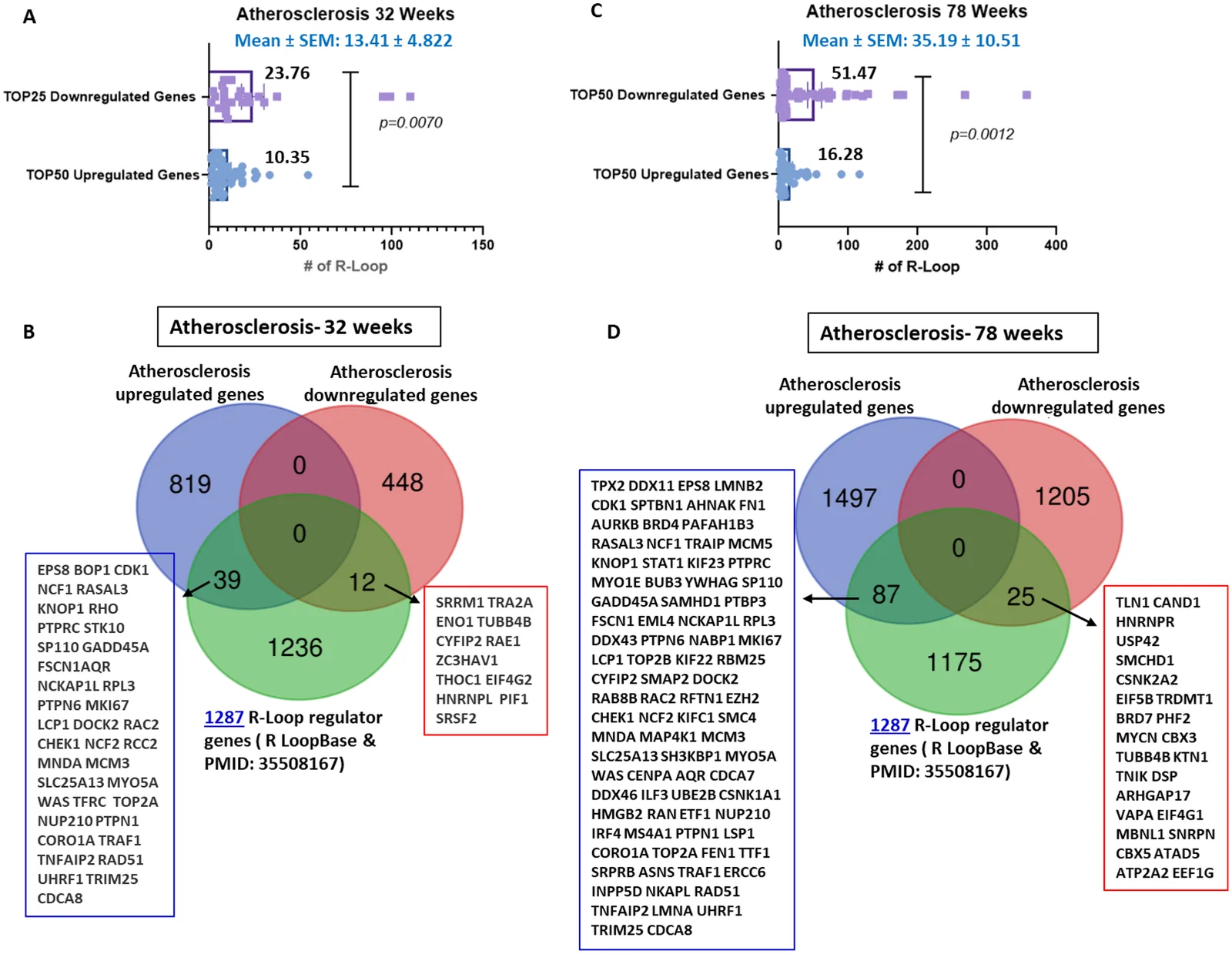
Increased R-loop numbers in downregulated genes during atherosclerosis progression. **(A)** In the 32-week high-fat diet (HFD)–induced atherosclerosis mouse model, the top 25 significantly downregulated genes displayed significantly higher R-loop numbers compared with the top 50 upregulated genes (*p* = 0.0070). **(B)** Venn diagram showing the overlap between known R-loop regulatory proteins and differentially expressed genes at 32 weeks. A total of 39 upregulated genes and 12 downregulated genes intersected with established R-loop regulators. **(C)** In the 78-week HFD-induced atherosclerosis model, the top 50 significantly downregulated genes similarly exhibited significantly higher R-loop numbers compared with the top 50 upregulated genes (*p* = 0.0012). **(D)** Venn diagram illustrating the overlap between R-loop regulatory proteins and differentially expressed genes at 78 weeks. Eighty-seven upregulated genes and 25 downregulated genes overlapped with known R-loop regulators. Transcriptomic datasets for HFD-induced atherosclerosis were obtained from GEO (GSE10000). R-loop regulatory proteins were compiled from R-loopBase and experimentally validated R-loop studies (23).

Building on our findings in the AAA model, we next investigated whether R-loop formation is altered in genes transcriptionally modulated during atherosclerosis progression in ApoE^−^/^−^ mouse aortas subjected to long-term hyperlipidemia induced by a high-fat diet (HFD) (4). Previously, we reported transcriptomic alterations in ApoE^−^/^−^ aortas at 32 and 78 weeks of HFD feeding, including significant upregulation of proinflammatory mediators (4). We hypothesized that the generations of R-loops in the genomic regions encoding for differentially expressed genes in atherosclerotic progression are modulated. To test this hypothesis, we used the same dataset (GSE10000) (24) to analyze R-loop numbers in the genomic regions of the top significantly upregulated and downregulated genes at each time point. At 32 weeks, the R-loop numbers in the genomic regions of the top 50 upregulated genes were significantly decreased compared to those of the top 25 downregulated genes (*p* < 0.007) (Figure 4A). At 78 weeks, the R-loop numbers in the genomic regions of the top 50 upregulated genes were significantly decreased compared to the top 50 downregulated genes (*p* < 0.0012) (Figure 4C). These findings suggest that like what was observed in the AAA model in the previous section, R-loop formation is decreased in genomic regions encoding transcriptionally upregulated genes during chronic atherosclerosis progression.

We next evaluated whether the expression of R-loop regulatory proteins changes during the atherosclerosis. Differential gene expression analysis of curated R-loop regulatory proteins from both the *Molecular Cell* review (52 genes) and R-loopBase (1,268 genes) was performed using the RNA-Seq datasets. At 32 weeks of HFD, 39 R-loop regulatory proteins were upregulated. The top five upregulated R-loop regulatory proteins include *CDK1* (2.94 log2FC, *p* = 0.0001), *BOP1* (1.56 log2FC, *p* = 0.0004), *NCF1* (1.3 log2FC, *p* = 0.003), *RASAL3* (1.3 log2FC, *p* = 0.02), and *EPS8* (1.2 log2FC, *p* = 0.003), while 12 were downregulated including the top five genes: *TUBB4B* (−2.06 log2FC, *p* = 0.0005), *SRRM1* (−1.93 log2FC, *p* = 0.01), *TRA2A* (−1.8 log2FC, *p* = 0.0009), *ENO1* (−1.3 log2FC, *p* = 0.002), and *CYFIP2* (−1.01 log2FC, *p* = 0.03) (Figure 4B). Enriched pathways among upregulated R-loop regulatory proteins included actin cytoskeleton organization, RAC2 GTPase cycle, regulation of T cell proliferation, cell population proliferation, and ATM signaling. Downregulated R-loop regulatory proteins were enriched in transport of mature mRNA derived from an intron-containing transcript, anti-viral mechanism by IFN-stimulated genes, and signaling by Rho GTPase.

At 78 weeks of HFD, 89 R-loop regulatory proteins were upregulated. The top five upregulated R-loop regulatory proteins include *CDK1 (*4.1 log2FC, *p* = 0.000004), *DDX11* (2.14 log2FC, *p* = 0.0004), *EPS8* (1.6 log2FC, *p* = 0.0006), *TPX2* (1.3 log2FC, *p* = 0.003), and *LMNB2* (1.29 log2FC, *p* = 0.002), while 25 were downregulated, including the top five: *USP42* (−2.36 log2FC, *p* = 0.01), *CAND1* (−2.12 log2FC, *p* = 0.02), *SMCHD1* (−2.02 log2FC, *p* = 0.002), *TLN1* (−1.27 log2FC, *p* = 0.0002), and *HNRNPR* (−1.03 log2FC, *p* = 0.003) (Figure 4D). The top 5 pathways for upregulated R-loop regulatory proteins included mitotic cell cycle, DNA metabolic process, regulation of cell cycle process, actin cytoskeleton organization, and Rho GTPase effectors and the top 5 pathways for downregulated R-loop regulatory proteins included epigenetic regulation of gene expression, translation, hemostasis, Wnt signaling pathway, and positive regulation of DNA metabolic process. Notably, many of the upregulated R-loop regulatory proteins at 32 and 78 weeks were associated with immune activation, cytoskeletal remodeling, and DNA damage responses, which align with previously reported increases in inflammatory and proliferative pathways during atherosclerosis (4). Taken together, these results provide new evidence that R-loop numbers are significantly reduced in the genomic regions of upregulated genes during late stages (32 and 78 weeks) of HFD-induced atherosclerosis in ApoE^−^/^−^ mice. The expression of R-loop regulatory proteins is dynamically altered over the course of disease progression, reflecting changes in transcriptional, immune, and cell cycle–related pathways. These findings extend our understanding of R-loop biology in chronic inflammatory vascular diseases and suggest that dysregulation of R-loop formation and resolution may contribute to the transcriptional reprogramming underlying vascular inflammation and plaque development in atherosclerosis.

### 2.3 R-loop numbers are reduced in genomic regions of upregulated genes in human NASH livers

We recently developed a novel metabolically healthy obesity mouse model by introducing microRNA-155 deficiency into the ApoE^−^/^−^ genetic background. Despite exhibiting features characteristic of non-alcoholic fatty liver disease (NAFLD), NASH, and metabolically healthy obesity, this model displayed significantly attenuated atherosclerosis (25). These findings highlight the pathogenic divergence between atherosclerosis and hepatic metabolic diseases, which, despite sharing common risk factors such as hyperlipidemia, may proceed via distinct molecular mechanisms (25–28). Emerging evidence suggests that the non-canonical inflammasome/caspase-11 contributes to HFD-induced NAFLD and NASH by promoting glycolysis, oxidative phosphorylation (OXPHOS), and pyroptosis in macrophages (29–31), potentially via trained immunity mechanism that enhance innate immune memory and inflammatory responsiveness (32–34).

**Figure 5:**
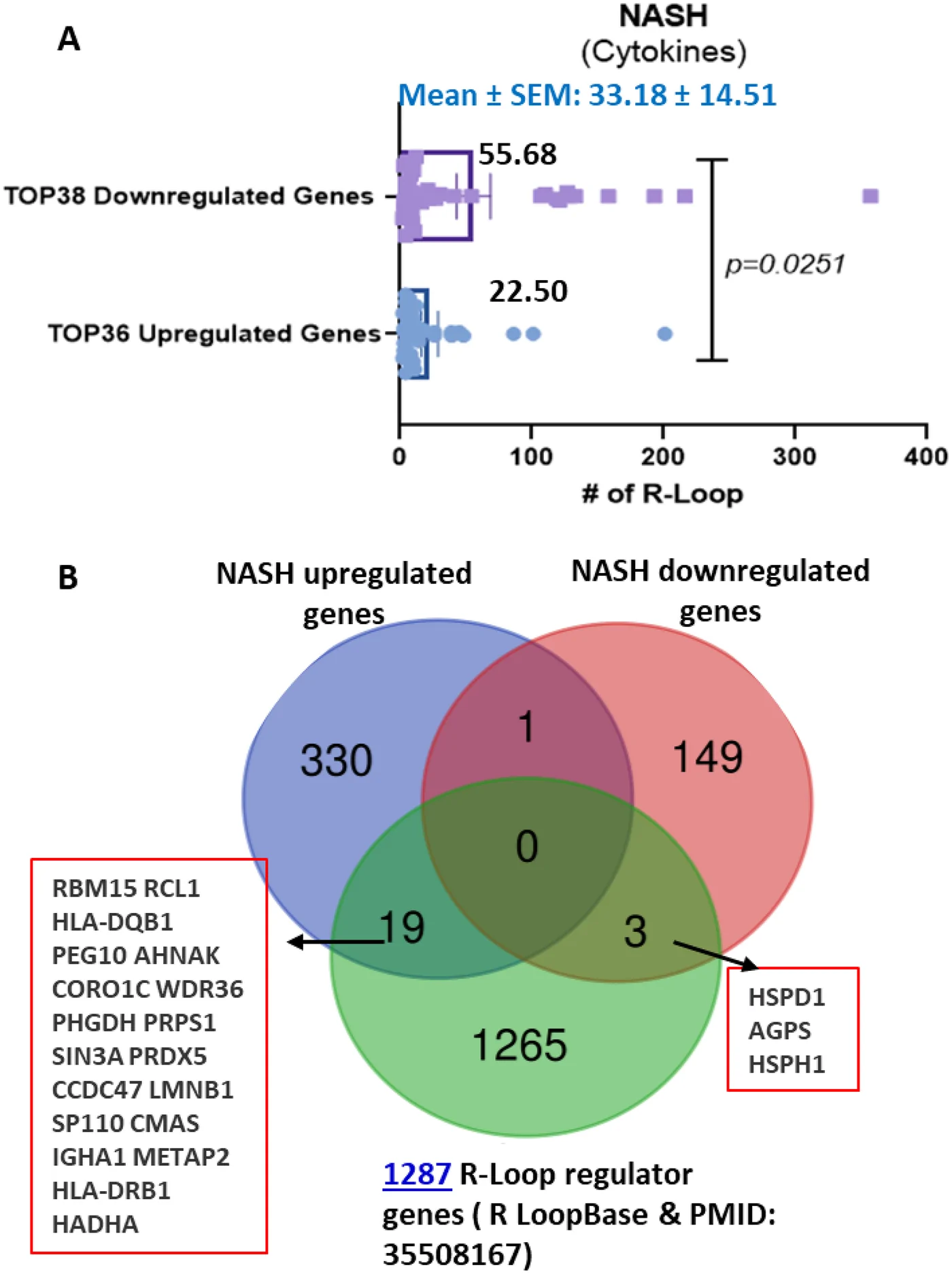
Enhanced R-loop accumulation in cytokine genes downregulated in non-alcoholic steatohepatitis (NASH). **(A)** Comparison of R-loop numbers between differentially expressed genes in NASH. The top 38 significantly downregulated genes exhibited significantly higher R-loop levels than the top 36 upregulated genes (*p* = 0.0251). **(B)** Venn diagram illustrating the intersection between differentially expressed genes in NASH and known R-loop regulatory proteins. Nineteen upregulated genes and three downregulated genes overlapped with established R-loop regulatory proteins. Transcriptomic data for NASH were obtained from the GEO dataset GSE63067, and the list of R-loop regulatory proteins was curated from R-loopBase and experimentally validated regulators reported in the literature (23).

We hypothesized that R-loop dynamics are altered in genomic regions encoding for differentially expressed genes during the progression of NASH. To test this, we analyzed publicly available transcriptomic datasets from NASH patients (GSE63067), obtained from the NCBI-GEOdatabase (https://www.ncbi.nlm.nih.gov/gds). As shown in Figure 5A, genomic regions encoding the top 36 upregulated cytokines in NASH livers exhibited a significant reduction in R-loop formation compared to regions associated with the top 38 downregulated cytokines (*p* < 0.0251). These findings suggest a preferential reduction in R-loop formation in transcriptionally activated regions during NASH progression.

To further investigate the potential regulation of R-loop homeostasis, we analyzed the expression levels of known R-loop regulatory proteins in NASH. As shown in Figure 5B, 19 R-loop regulatory proteins were upregulated including top five: *PEG10 (*1.14 log2FC, *p* = 0.03), *RCL1* (1.04 log2FC, *p* = 0.003), *HADHA* (0.9 log2FC, *p* = 0.02), *HLA-DQB1* (0.9 log2FC, *p* = 0.03), and *CORO1C* (0.8 log2FC, *p* = 0.03), while and 3 were downregulated in NASH livers, including *HSPD1* (−1.2 log2FC, *p* = 0.005), *AGPS* (−0.6 log2FC, *p* = 0.005), and *HSPH1* (−0.7 log2FC, *p* = 0.04). Pathway enrichment analysis revealed that the upregulated R-loop regulatory proteins were predominantly involved in processes such as heterochromatin formation, antigen receptor-mediated signaling pathway, regulation of myeloid cell differentiation, endomembrane system organization, and cellular homeostasis. Collectively, these findings demonstrate that R-loop formation is selectively reduced in genomic regions corresponding to upregulated genes in NASH, consistent with patterns previously observed in chronic vascular inflammatory diseases such as atherosclerosis and AAA.

These results support a potential shared mechanism linking altered R-loop dynamics to transcriptional regulation in diverse chronic inflammatory diseases.

### 2.4 R-loop numbers are reduced in genomic regions of upregulated genes in MSU-stimulated lymphatic endothelial cells (LECs)

**Figure 6:**
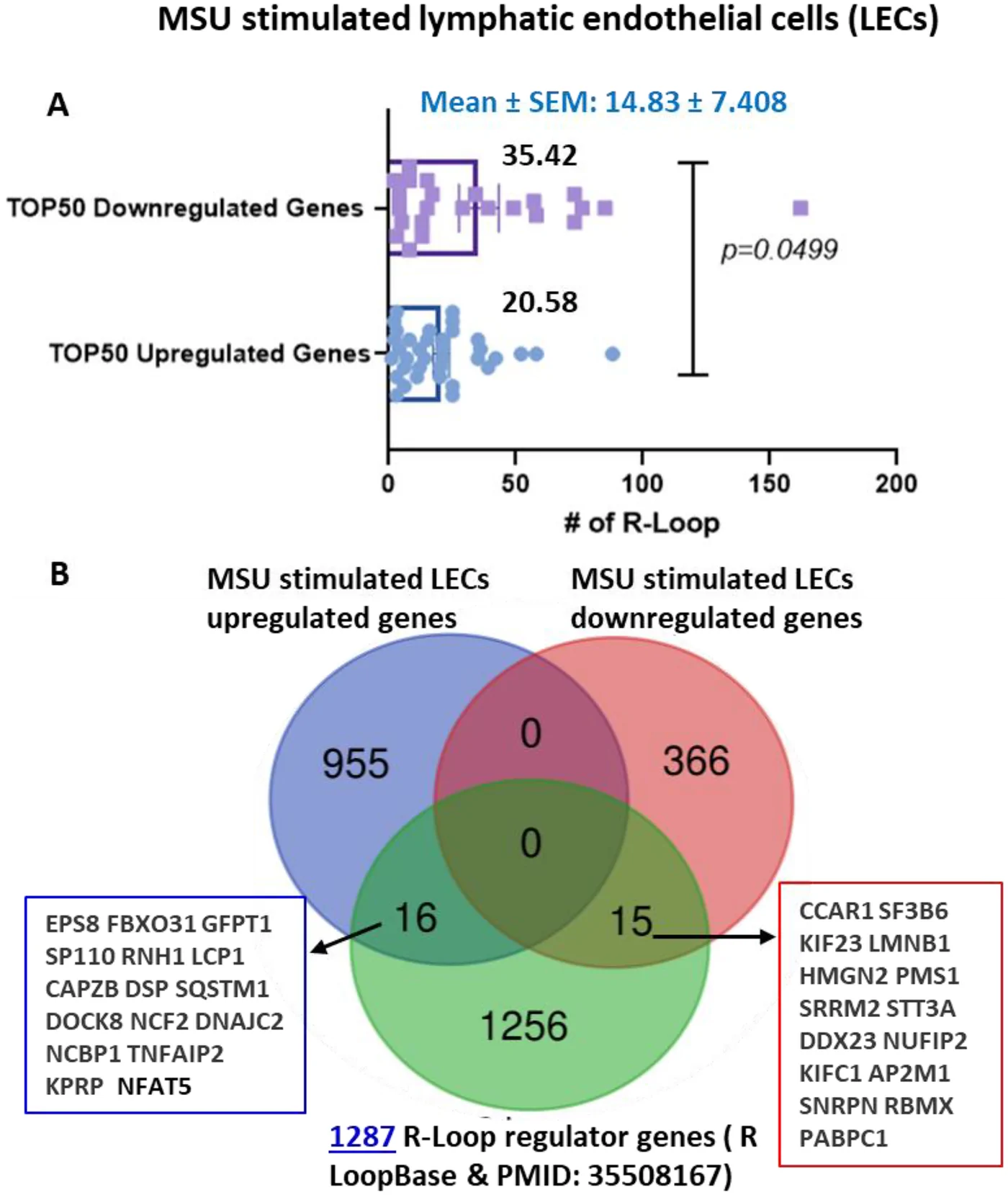
Increased R-loop numbers in downregulated genes in lymphatic endothelial cells (LECs) stimulated with monosodium urate (MSU). **(A)** Comparison of R-loop numbers between differentially expressed genes in MSU-stimulated LECs. The top 50 significantly downregulated genes exhibited significantly higher R-loop numbers than the top 50 upregulated genes (*p* = 0.0499). **(B)** Venn diagram illustrates the overlap between differentially expressed genes in MSU-stimulated LECs and known R-loop regulatory proteins. A total of 16 upregulated genes and 15 downregulated genes intersected with established R-loop regulators. Transcriptomic data for MSU-stimulated LECs were obtained from the GEO dataset GSE199950, and the list of R-loop regulatory proteins was curated from R-loopBase and experimentally validated regulators reported in the literature (23).

Gout is well known to be associated with other metabolic disorders such as dyslipidemia, obesity, and type 2 diabetes, particularly in dyslipidemia with elevated low-density lipoprotein (LDL) cholesterol and hypertriglyceridemia (35). Elevated serum uric acid levels, a hallmark of gout, contributes to increased LDL cholesterol and triglyceride levels (36, 37). Interestingly, pharmacological reduction of uric acid with allopurinol has been shown to reduce triglyceride accumulation by decreasing intracellular uric acid levels (35). Previously, we proposed a new concept that ECs possess innate immune functions (17, 38–41), suggesting they may actively participate in inflammatory responses to endogenous danger signals. To investigate how a metabolite-derived danger-associated molecular pattern (DAMP) (16)—specifically MSU crystals, a key driver of gouty inflammation—modulates gene regulation in ECs, we hypothesized that R-loop dynamics are altered in LECs in response to MSU stimulation. To test this hypothesis, we analyzed transcriptomic data from dataset GSE199950 in the NIH NCBI GEOdatabase, where LECs were stimulated with MSU to model sterile inflammation relevant to gout. As shown in Figure 6A, R-loop numbers were significantly reduced in genomic regions of the top 50 upregulated genes, compared to those associated with the top 50 downregulated genes (*p* < 0.0499). This suggests that transcriptional activation in response to MSU is associated with reduced R-loop formation.

Next, we examined the expression profiles of known R-loop regulatory genes. In MSU-treated LECs, 16 R-loop regulatory genes were upregulated including *FBXO31* (1.8 log2FC, *p* = 0.0000005), *GFPT1* (1.6 log2FC, *p* = 0.0001), *SP110* (1.3 log2FC, *p* = 0.0008), *EPS8* (1.13 log2FC, *p* = 0.000002), and *RNH1* (1.12 log2FC, *p* = 0.02), while 15 were downregulated, including *LMNB1* (−1.54 log2FC, *p* = 0.0009), *CCAR1* (−1.4 log2FC, *p* = 0.0005), *SF3B6* (−1.32 log2FC, *p* = 0.02), *HMGN2* (−1.19 log2FC, *p* = 0.00004), *KIF23* (−1.13 log2FC, *p* = 0.0004) (Figure 6B). Pathway enrichment analysis revealed that the upregulated R-loop regulator genes were predominantly involved in cellular response to stress, regulation of actin filament-based process, regulation of small GTPase mediated signal transduction, Rho GTPase cycle, and transcription by RNA polymerase II. Conversely, downregulated R-loop regulators were enriched in pathways related to mRNA splicing–major pathway and membrane trafficking.

Previous studies have demonstrated that MSU crystal-induced release of interleukin-1β (IL-1b) is dependent on activation of Toll-like receptor 4 (TLR4) and transient receptor potential vanilloid 1 (TRPV1) (42); and that at the base of the inflammatory cascade, stimulated by MSU crystals, there are many complex cellular mechanisms mainly involving the NOD-like receptors (NLR) family pyrin domain containing 3 (NLRP3) inflammasome as we reported (16, 43, 44), the hallmark of autoinflammatory syndromes (45). Taken together, these findings demonstrate for the first time that MSU-induced signaling via TLR4, TRPV1, and cytosolic NLRP3 inflammasome pathways is associated with decreased R-loop formation in genomic regions encoding upregulated innate immune and proinflammatory genes in LECs. This underscores a novel layer of epigenetic regulation in the inflammatory response to metabolic DAMPs and supports the concept of ECs as key innate immune effectors in sterile inflammatory diseases such as gout.

### 2.5 R-loop numbers are decreased in genomic regions of upregulated innate immune genes in influenza virus-infected human umbilical vein endothelial cells (HUVECs)

**Figure 7:**
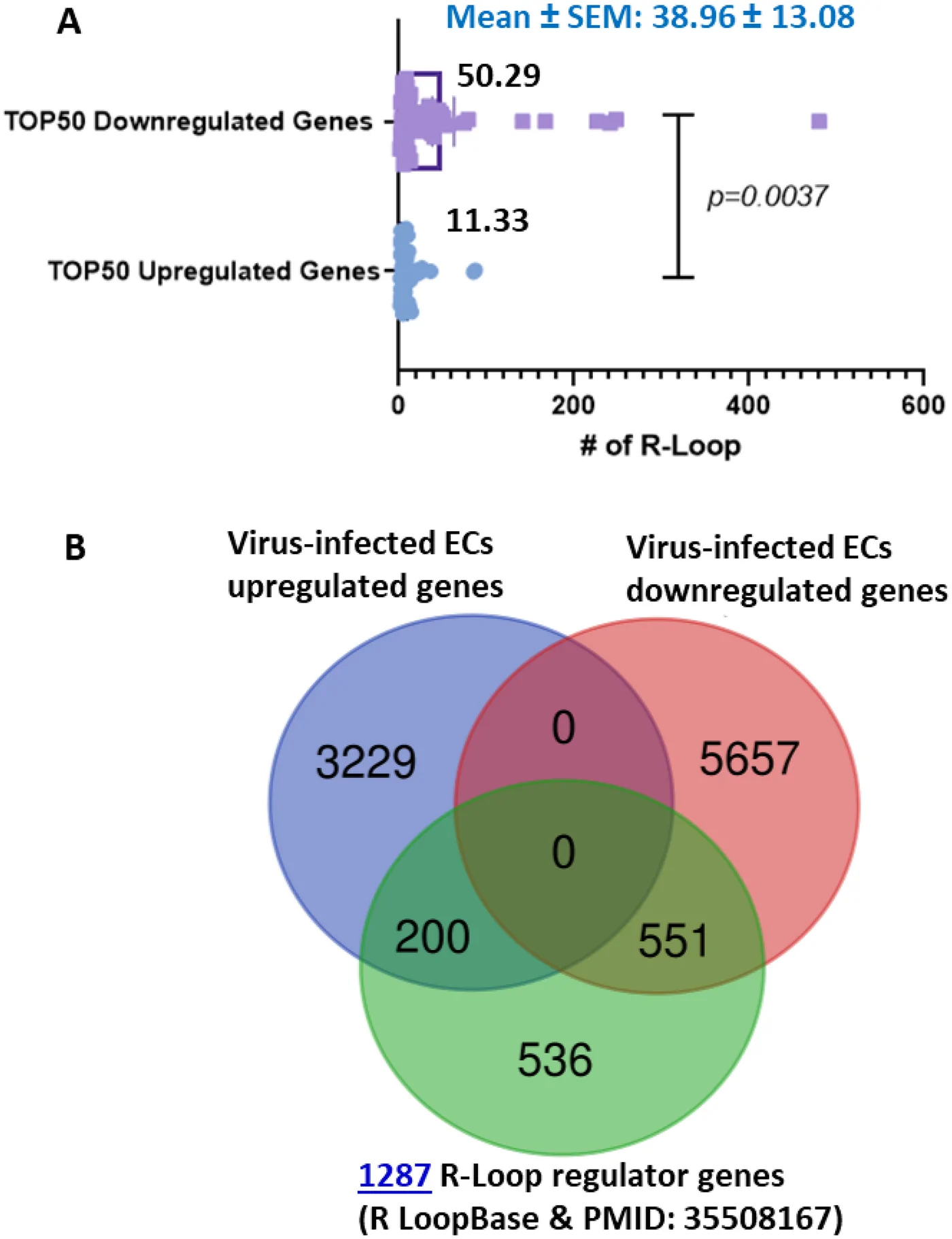
Increased R-loop numbers in downregulated genes in human umbilical vein endothelial cells (HUVECs) infected with influenza virus. **(A)** Comparison of R-loop numbers between differentially expressed genes in HUVECs following influenza virus infection. The top 50 significantly downregulated genes exhibited significantly higher R-loop numbers than the top 50 upregulated genes (*p* = 0.0037). **(B)** Venn diagram illustrates the overlap between differentially expressed genes in influenza-infected HUVECs and known R-loop regulatory proteins. A total of 200 upregulated genes and 551 downregulated genes intersected with established R-loop regulators. Transcriptomic data were obtained from the GEO dataset GSE59226, and the list of R-loop regulatory proteins was curated from R-loopBase and experimentally validated regulators reported in the literature (23).

Recent studies indicate that both viral replication and innate immune responses are modulated by cellular cholesterol levels. Specifically, cholesterol depletion suppresses both processes, suggesting that viruses and host immune responses share a dependence on elevated intracellular cholesterol. This insight supports the development of antiviral strategies aimed at reducing cellular cholesterol content (46). Building on our prior investigations into R-loop regulation in response to cholesterol and hyperlipidemia-derived metabolic stimuli and sterile inflammatory signals, such as DAMPs (e.g., MSU crystals), we next sought to determine whether PAMPs—specifically, influenza virus infection—modulate R-loop formation in vascular ECs. We hypothesized that influenza virus infection alters R-loop dynamics in human HUVECs. As shown in Figure 7A, the number of R-loops in the genomic regions encoding the top 50 upregulated genes was significantly reduced compared to those associated with the top 50 downregulated genes (*p* < 0.0037).

To explore potential regulatory mechanisms, we examined changes in the expression of R-loop regulatory genes. In total, 200 R-loop regulators were upregulated including *NCL* (3.4 log2FC, *p* = 0.000006), *SFPQ* (2.61 log2FC, *p* = 0.000001), ZNF346 (2.16 log2FC, *p* = 0.00001), *RAD52* (2.1 log2FC, *p* = 0.00009), and *SF3B1* (1.02 log2FC, *p* = 0.0004), while 551 were downregulated in influenza-infected HUVECs, including *BRD4* (−2.88 log2FC, *p* = 0.000009), *PRMT5* (−2.3 log2FC, *p* = 0.000003), *ERCC5* (−2.3 log2FC, *p* = 0.000003), *ADAR* (−1.9 log2FC, *p* = 0.00001), *ATM* (−1.23 log2FC, *p* = 0.0006) (Figure 7B). Pathway enrichment analysis revealed that the upregulated R-loop regulators were primarily involved in RNA metabolism, ribonucleoprotein complex biogenesis, regulation of mRNA metabolic process, Nop56-associated pre-rRNA complex, and chromatin remodeling. Conversely, downregulated R-loop regulators were enriched in pathways including ribonucleoprotein complex biogenesis, DNA metabolic process, regulation of DNA metabolic process, cell cycle, and ribosome biogenesis.

Influenza virus infection was included in this study because it has been reported to increase atherosclerosis (47, 48). Therefore, although influenza is an infectious disease model, its inclusion is relevant to the cardiovascular focus of this study. In this context, the influenza dataset supports the concept that the relationship between altered R-loop numbers and proinflammatory gene regulation may also be observed in inflammatory conditions that have documented links to atherosclerotic disease.

It is well-established that the human upper respiratory tract epithelium expresses sialylated glycan receptors terminated by *α*2-6-linked sialic acid residues, which serve as entry points for influenza A virus via hemagglutinin (HA) binding (49). Influenza virus engagement with these receptors initiates host cell signaling cascades involving TFs such as STATs (50) and non-canonical pattern recognition pathways, including Ca²⁺/calmodulin-dependent protein kinase II (CaMKII)-mediated activation of retinoic acid-inducible gene I (RIG-I) (51). Together, these findings demonstrate for the first time that influenza virus infection reduces R-loop numbers in genomic regions encoding upregulated genes in HUVECs. This response likely reflects a virus-induced reprogramming of host transcriptional and epigenetic machinery, adding a novel layer of complexity to how ECs, functioning as innate immune effectors, respond to PAMPs during viral infections.

### 2.6 The ratios of the R-loop numbers in genomic regions encoded for the disease-upregulated genes over that of the disease-downregulated genes are higher in advanced disease stages than that of earlier stages of diseases

We hypothesized that the ratios of the R-loop numbers in genomic regions encoding disease-upregulated genes vs. downregulated genes increase with disease progression. To investigate this, we calculated the mean R-loop number ratios in genomic regions corresponding to the top differentially expressed genes across multiple disease models and time points. As summarized in Table 2, the ratios of mean R-loop numbers (downregulated genes vs. upregulated genes) in genomic regions were calculated for each disease and stage: AAA 14 days (2.25), AAA 28 days (3.42), atherosclerosis 32 weeks (2.3), atherosclerosis 78 weeks (3.16), NASH (2.47), MSU-stimulated LECs (1.72), and influenza virus infected ECs (4.44). Notably, AAA progression from 14 to 28 days showed an increase in the R-loop ratio from 2.25 to 3.42, while atherosclerosis progression from 32 to 78 weeks showed an increase from 2.3 to 3.16. These increases suggest that R-loop reduction in disease-upregulated genes become more pronounced over time, particularly in chronic inflammatory settings.

These trends correlate with previously published transcriptomic data on innate immune gene activation (4). Specifically, the percentage of upregulated innate immune genes increased from 2.4% to 10.2% in AAA (14 to 28 days) and from 11.0% to 17.7% in atherosclerosis (32 to 78 weeks), indicating that innate immune activation parallels the progressive reduction of R-loops in upregulated genes.

To further investigate the mechanism behind decreased R-loop numbers in upregulated gene loci, we examined whether R-loop regulatory proteins upregulated in disease conditions are enriched in immune-related pathways. As summarized in Table 3, AAA (28 days) upregulated R-loop regulators were significantly enriched in immune pathways, including cell population proliferation, microglia pathogen phagocytosis, B cell receptor signaling, cytokine signaling in immune system, leukocyte proliferation, and immune effector process; atherosclerosis (32 weeks) upregulated R-loop regulators were enriched in T cell proliferation, cell population proliferation, adherens junction, and interferon signaling; atherosclerosis (78 weeks) upregulated R-loop regulators were enriched in immune pathways included mitotic cell cycle, regulation of cell cycle, regulation of response to biotic stimulus, and regulation of cell cycle phase transition; NASH upregulated R-loop regulators were enriched in a few immune pathways included antigen receptor-mediated signaling, regulation of myeloid cell differentiation, and cellular homeostasis; MSU stimulated LECs upregulated R-loop regulators were enriched in a few immune pathways included cellular response to stress. In contrast, very few immune pathways were enriched in downregulated R-loop regulators, with only a single pathway (e.g., *antiviral IFN-stimulated gene response*) identified in downregulated regulators in atherosclerosis (78 weeks). For influenza virus-infected ECs, although both up- and downregulation of numerous R-loop regulators were observed, the top associated pathways were not directly immune-related, and were probably related to R-loop biogenesis, processing, and resolution, consistent with viral transcriptional hijacking mechanisms.

**Table 2:** Summary of R-loop numbers in upregulated and downregulated genes across multiple disease models.

Disease conditions	Upregulated genes	Downregulated genes		
	Mean of R loops	Mean of R loops	Mean ± SEM	Ratio (Down/Up)
AAA 14 days	21	47.23	26.23 ± 9.795	2.25
AAA 28 days	15.64	53.47	37.83 ± 11.84	3.42
Atherosclerosis 32 weeks	10.35	23.76	13.41 ± 4.822	2.3
Atherosclerosis 78 weeks	16.28	51.47	35.19 ± 10.51	3.16
NASH	22.5	55.68	33.18 ± 14.51	2.47
MSU stimulated LECs	20.58	35.42	14.83 ± 7.408	1.72
Influenza virus infected ECs	11.33	50.29	38.96 ± 13.08	4.44

The table presents a comparative overview of R-loop numbers in upregulated and downregulated genes identified from RNA-seq datasets across five pathological conditions, including AAA, atherosclerosis, NASH, MSU-stimulated LECs modeling gouty arthritis (GA), and Influenza virus-infected ECs. Across all conditions, R-loop signal analysis consistently revealed greater R-loop numbers in the transcriptionally downregulated gene sets compared to the upregulated counterparts.

**Table 3:** Pathway enrichment analysis of genes with differential R-loop accumulation across inflammatory and cardiovascular disease models.

Datasets	Top 10 pathways of the upregulated R loops (Log10 (*P*-value) <−3.5	Top 10 pathways of the downregulated R loops (Log10 (P) < −3.5
AAA 14 days	-	mRNA metabolic process Cellular response to chemical stress RNA localization Metabolism of RNA Regulation of mRNA metabolic process Regulation of cell cycle process Positive regulation of cellular component biogenesis DNA repair Heterochromatin formation ATP-dependent chromatin remodeling
AAA 28 days	Cell population proliferation RAC1 GTPase cycle Microglia pathogen phagocytosis pathway B cell receptor signaling Cortical cytoskeleton organization Cytokine Signaling in Immune system Leukocyte proliferation Immune effector process ChREBP activates metabolic gene expression DNA IR damage and cellular response via ATR	-
Atherosclerosis—32 weeks	Actin cytoskeleton organization RAC2 GTPase cycle Regulation of T cell proliferation cell population proliferation ATM signaling Regulation of DNA recombination Regulation of protein-containing complex assembly Cell Cycle Adherens junction Interferon Signaling	Transport of Mature mRNA derived from an Intron-Containing Transcript Antiviral mechanism by IFN-stimulated genes Signaling by Rho GTPases
Atherosclerosis—78 weeks	Mitotic cell cycle DNA metabolic process Regulation of cell cycle process Actin cytoskeleton organization RHO GTPase Effectors Regulation of DNA metabolic process Regulation of response to biotic stimulus SUMOylation of DNA replication proteins Negative regulation of organelle organization Regulation of cell cycle phase transition	Epigenetic regulation of gene expression Translation Hemostasis Wnt signaling pathway Positive regulation of DNA metabolic process
Human NASH	Heterochromatin formation Antigen receptor-mediated signaling pathway Regulation of myeloid cell differentiation Endomembrane system organization Cellular homeostasis	-
MSU stimulated LECs	Cellular responses to stress Regulation of actin filament-based process Regulation of small GTPase mediated signal transduction RHO GTPase cycle Transcription by RNA polymerase II	mRNA Splicing—Major Pathway Membrane Trafficking
Influenza virus infected human umbilical vein endothelial cells.	Metabolism of RNA Ribonucleoprotein complex biogenesis Regulation of mRNA metabolic process Nop56p-associated pre-rRNA complex Chromatin remodeling Protein-RNA complex assembly RNA Polymerase II Transcription Termination Regulation of DNA metabolic process Regulation of translation DNA metabolic process	Ribonucleoprotein complex biogenesis DNA metabolic process Regulation of DNA metabolic process Cell Cycle Ribosome biogenesis Regulation of cell cycle process Chromatin remodeling Regulation of chromosome organization Regulation of translation DNA IR damage and cellular response via ATR

Metascape pathway enrichment analysis was performed on genes exhibiting significant upregulation or downregulation of R-loop signals in five pathological contexts: AAA, atherosclerosis, NASH, MSU-stimulated LECs modeling gouty arthritis (GA), and Influenza virus-infected ECs. This analysis identified the top enriched biological processes and pathways associated with R-loop dysregulation in both upregulated and downregulated gene sets, providing insight into disease-specific mechanisms potentially driven by R-loop accumulation.

Taken together, these findings demonstrate that the ratios of the R-loop numbers in the genomic regions encoding disease-upregulated genes vs. downregulated genes increase with disease progression in models of AAA and atherosclerosis; R-loop regulatory proteins upregulated during disease progression are enriched in immune and inflammatory pathways, supporting the concept of immune-driven R-loop remodeling. These results provide the first evidence that decreased R-loop numbers in genomic regions encoding disease-upregulated genes are a consistent feature across models of metabolic inflammation including AAA, atherosclerosis, NASH, and MSU-induced endothelial activation. This decrease is likely driven by innate immune reprogramming of R-loop regulators, which may serve as both sensors and effectors of chronic inflammation.

These findings reveal a potentially unifying epigenetic–immune axis in chronic inflammatory diseases and open avenues for targeting R-loop dynamics and their regulators for therapeutic intervention in vascular and metabolic pathologies.

### 2.7 NRF2 promotes the expressions of 10 and inhibits 17 R-loop regulators; and the upregulation of these 8 NRF2^−^/^−^ upregulated (pro-ROS) R-loop regulators and downregulation of 8 NRF2^−^/^−^ downregulated (anti-ROS) R-loop regulators in metabolic diseases and influenza viral infection may contribute to diseases pathogenesis

**Figure 8:**
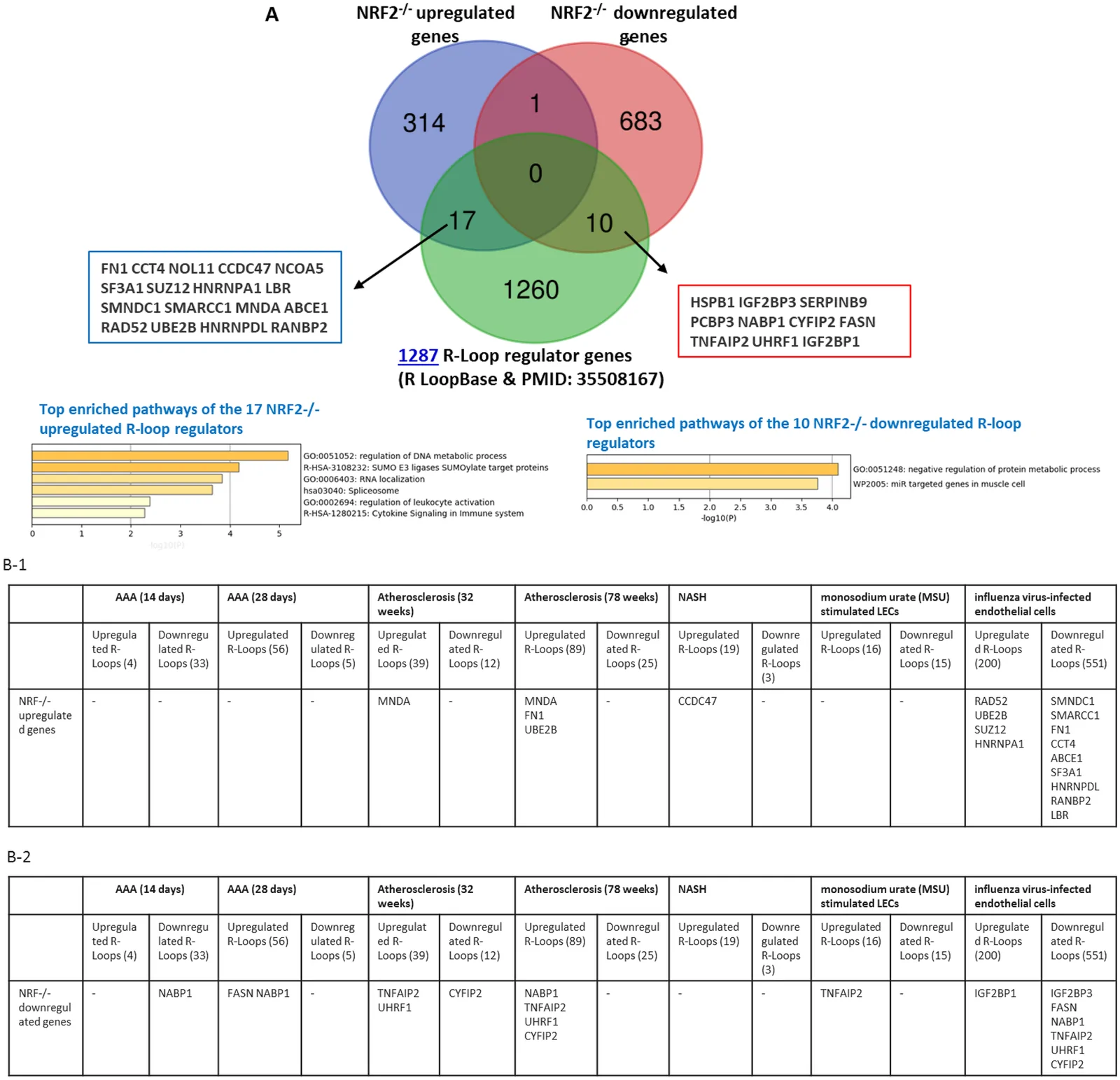
NRF2-dependent regulation of R-loop regulatory genes and their expression patterns across inflammatory disease datasets. **(A)** Transcriptomic analysis of NRF2-deficient models revealed that the antioxidant transcription factor NRF2 positively regulates 10 R-loop regulatory genes (downregulated under NRF2-deficient conditions) and negatively regulates 17 R-loop regulatory genes (upregulated under NRF2-deficient conditions). Pathway enrichment analysis showed that NRF2-activated R-loop regulators were enriched in two pathways—negative regulation of protein metabolic processes and miRNA-targeted genes in muscle cells—whereas NRF2-repressed R-loop regulators were enriched in six pathways, including regulation of DNA metabolic processes, SUMO E3 ligase–mediated SUMOylation of target proteins, RNA localization, spliceosome function, regulation of leukocyte activation, and cytokine signaling in the immune system. These findings highlight NRF2 as a modulator of distinct subsets of R-loop regulators with implications for genomic stability, immune function, and post-transcriptional RNA regulation. (B-1) Seven NRF2-inhibited R-loop regulatory genes (i.e., genes upregulated upon NRF2 deficiency or knockout)—MNDA, FN1, UBE2B, CCDC47, RAD52, SUZ12, and HNRNPA1—were consistently upregulated in five of eight inflammatory disease datasets, consistent with previous evidence that pro-oxidative stress regulators are frequently elevated in inflammatory conditions (17). (B-2) Seven NRF2-promoted R-loop regulatory genes (i.e., genes downregulated upon NRF2 knockout)—NABP1, CYFIP2, IGF2BP3, FASN, TNFAIP2, and UHRF1—were downregulated in three of eight disease datasets. Notably, eight NRF2-promoted R-loop regulators (FASN, NABP1, TNFAIP2, UHRF1, CYFIP2, IGF2BP1) were also upregulated in five of eight diseases, suggesting the presence of compensatory or context-dependent regulatory mechanisms.

We have previously reported that the antioxidant transcription factor nuclear factor erythroid 2 (NFE2)-related factor 2 (NRF2; encoded by *NFE2L2*) plays a critical role in regulating ROS, innate immune responses, trained immunity, EC activation and death, and thromboembolism (17). Given our findings of reduced R-loop numbers at genomic regions associated with genes upregulated in metabolic inflammations and the observed modulation of R-loop regulators, we hypothesized that NRF2 may regulate the R-loop regulators, potentially influencing disease outcomes. To test this hypothesis, we analyzed transcriptomic data from NRF2-deficient (NRF2^−^/^−^) models (GSE71695) and identified 27 R-loop regulators whose expression is significantly modulated by NRF2. Of these, 10 R-loop regulators were downregulated in NRF2^−^/^−^ models, including *HSPB1* (Heat Shock Protein Family B (Small) Member 1), *IGF2BP3* (Insulin Like Growth Factor 2 MRNA Binding Protein 3), *SERPINB9* (Serpin Family B Member 9), *PCBP3* (Poly(RC) Binding Protein 3), *NABP1* (Nucleic Acid Binding Protein 1), *CYFIP2* (Cytoplasmic FMR1 Interacting Protein 2), *FASN* (Fatty Acid Synthase), *TNFAIP2* (TNF Alpha Induced Protein 2), *UHRF1* (Ubiquitin Like With PHD And Ring Finger Domains 1), and *IGF2BP1* (Insulin Like Growth Factor 2 MRNA Binding Protein 1), suggesting that NRF2 positively regulates their expression. These genes may function as an antioxidant (anti-ROS) R-loop regulators under NRF2 control. Conversely, 17 R-loop regulators were upregulated in NRF2^−^/^−^, including *FN1* (Fibronectin 1), *CCT4* (Chaperonin Containing TCP1 Subunit 4), *NOL11* (Nucleolar Protein 11), *CCDC47* (Coiled-Coil Domain Containing 47), *NCOA5* (Nuclear Receptor Coactivator 5), *SF3A1* (Splicing Factor 3a Subunit 1), *SUZ12* (SUZ12 Polycomb Repressive Complex 2 Subunit), *HNRNPA1* (Heterogeneous Nuclear Ribonucleoprotein A1), *LBR* (Lamin B Receptor), *SMNDC1* (Survival Motor Neuron Domain Containing 1), *SMARCC1* (SWI/SNF Related BAF Chromatin Remodeling Complex Subunit C1), *MNDA* (Myeloid Cell Nuclear Differentiation Antigen), *ABCE1* (ATP Binding Cassette Subfamily E Member 1), *RAD52* (RAD52 Homolog, DNA Repair Protein), *UBE2B* (Ubiquitin Conjugating Enzyme E2 B), *HNRNPDL* (Heterogeneous Nuclear Ribonucleoprotein D Like), and *RANBP2* (RAN Binding Protein 2), indicating they may be normally repressed by NRF2 and may serve as pro-oxidant (pro-ROS) R-loop regulators (Figure 8A).

To further assess whether the identified RLPs may be directly regulated by NRF2, we cross-referenced the 27 NRF2-associated RLPs with publicly available NRF2/NFE2L2 ChIP-seq target-gene datasets *(*https://chip-atlas.dbcls.jp/data/hg38/target/NFE2L2.1.html*)*. This analysis showed that 21 of the 27 RLPs exhibited promoter-proximal NRF2 binding evidence, including LBR, CCT4, CCDC47, HNRNPA1, SMARCC1, ABCE1, RANBP2, UBE2B, NABP1, HSPB1, RAD52, NOL11, SMNDC1, FN1, SUZ12, FASN, HNRNPDL, NCOA5, SF3A1, IGF2BP3, and CYFIP2. These findings support the concept that NRF2 may regulate a substantial fraction of the R-loop regulatory network identified in this study. However, because public ChIP-seq datasets are derived from multiple cell systems and experimental conditions, these data should be interpreted as supportive evidence of potential direct regulation rather than definitive proof in disease-relevant vascular, hepatic, or inflammatory primary cells.

We next examined the expression of these 27 NRF2-modulated R-loop regulators in the context of metabolic diseases and influenza viral infection. As shown Figure 8B, four NRF2^−^/^−^-upregulated (pro-ROS) R-loop regulators were also upregulated in metabolic inflammatory conditions including those in the red boxes such as *MNDA* (atherosclerosis 32 weeks), *MNDA, FN1, UBE2B* (atherosclerosis 78 weeks), and *CCDC47* (NASH). Additionally, four NRF2^−^/^−^ upregulated (pro-ROS) regulators—*RAD52*, *UBE2B*, *SUZ12*, and *HNRNPA1*—were also upregulated in influenza virus-infected human ECs. The consistent upregulation of these eight pro-ROS R-loop regulators in both metabolic inflammation and viral infection suggests their potential contribution to disease pathogenesis. We also observed that two NRF2^−^/^−^ downregulated (anti-ROS) R-loop regulators in the blue boxes —*NABP1* and *CYFIP2*—were downregulated in AAA 14 days and atherosclerosis 32 weeks, respectively. Furthermore, six NRF2^−^/^−^ downregulated (anti-ROS) R-loop regulators including those in the blue box—*IGF2BP3, FASN, NABP1, TNFAIP2, UHRF1*, and *CYFIP2—*were also downregulated in influenza virus-infected ECs. The downregulation of these eight anti-ROS R-loop regulators may impair antioxidant defense mechanisms and promote inflammation and tissue damage during disease. Of note, nine NRF2^−^/^−^ upregulated pro-ROS R-loop regulators were found to be downregulated in influenza-infected ECs (outside of the red box), suggesting a limited role in the pathogenesis of influenza. In addition, ten NRF2^−^/^−^ downregulated anti-ROS R-loop regulators were paradoxically upregulated during inflammatory conditions, which may reflect a compensatory anti-inflammatory response. This is consistent with our previous findings showing that anti-inflammatory cytokines such as IL-35 (52–55) and IL-10 (56) are upregulated in response to inflammatory stimuli.

Taken together, these data demonstrate that NRF2 positively regulates the expression of 10 putative anti-ROS R-loop regulators and negatively regulates 17 pro-ROS R-loop regulators. The dysregulation of these genes—particularly the upregulation of 8 pro-ROS and downregulation of 8 anti-ROS R-loop regulators—in metabolic diseases and influenza virus infection may contribute to the pathogenesis of these conditions.

### 2.8 R-loop regulators are shared with mitochondrial and cellular ROS regulators, indicating mutual regulation

**Figure 9:**
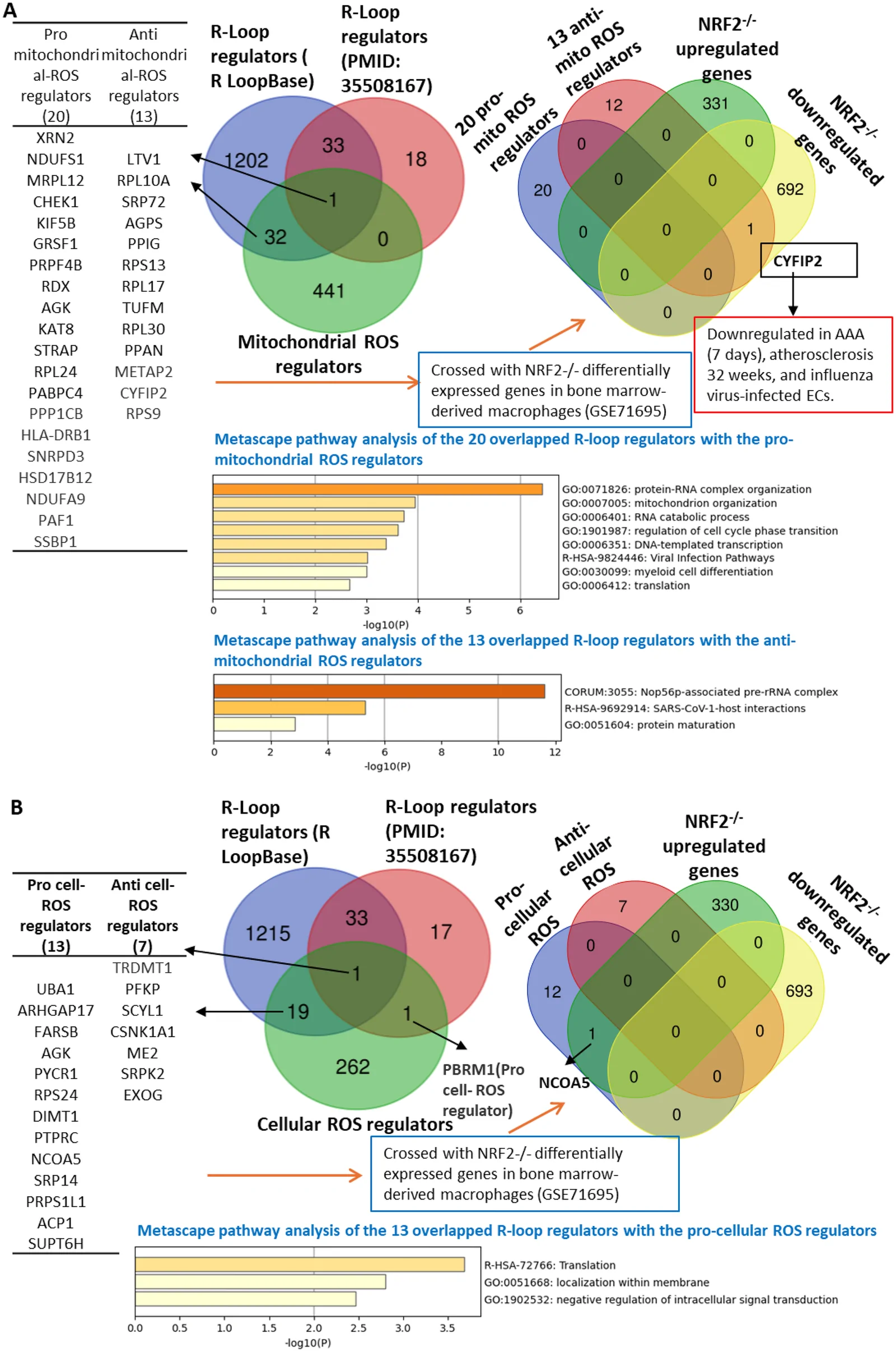
Functional interplay between R-loop regulatory genes and mitochondrial or cellular ROS regulators. **(A)** Venn diagram analysis comparing R-loop regulators with CRISPR interference (CRISPRi)-identified mitochondrial reactive oxygen species (mtROS) regulators revealed 20 overlapping pro-mtROS/R-loop regulators and 13 anti-mtROS/R-loop regulators. Pathway enrichment analysis showed that pro-mtROS/R-loop regulators were significantly enriched in protein–RNA complex organization, mitochondrial organization, RNA catabolic process, regulation of cell cycle phase transition, and DNA-templated transcription, whereas anti-mtROS/R-loop regulators were enriched in Nop56p-associated pre-rRNA complex, SARS-CoV-1 host interaction, and protein maturation pathways. Integration with the NRF2^−^/^−^ transcriptomic dataset (GSE71695) revealed that the anti-mtROS regulator CYFIP2 is transcriptionally promoted by NRF2. **(B)** Venn diagram analysis comparing R-loop regulators with 283 cellular ROS regulators identified 14 overlapping pro-cellular ROS/R-loop regulators (13 from the main table plus PBRM1) and 7 anti-cellular ROS/R-loop regulators. Pathway enrichment analysis of pro-cellular ROS/R-loop regulators revealed enrichment in translation, membrane localization, and negative regulation of intracellular signal transduction. Notably, the pro-cellular ROS regulator NCOA5 is transcriptionally repressed by NRF2, consistent with its role as an NRF2-inhibited pro-oxidant gene.

We recently proposed a conceptual framework in which ROS act as an integrated sensor network that monitor cellular homeostasis and alarming stresses in organelle metabolic processes (57). We and others have reported that hyperlipidemia (43, 54, 58–60), hyperlipidemia combined with angiotensin II (Ang II, the context of AAA) (4, 20), NASH (31, 32), MSU-induced gouty arthritis (61), uremic toxins and chronic kidney disease (CKD) (34, 60, 62, 63), as well as influenza virus infection (64), all induce metabolic reprogramming (29), leading to increased production of mitochondrial ROS (mitoROS) (65, 66) and cellular ROS (57). Recently, Bennett et al. conducted a comprehensive systems-level CRISPR interference (CRISPRi) screening and identified 474 mitoROS regulators and 283 cellular ROS regulators (67). Based on these findings, we hypothesized that R-loop regulators may partially overlap and functionally interact with mitochondrial and cellular ROS regulators, contributing to mutual regulatory pathways linking nuclear genome stability and redox signaling. To test this hypothesis, we performed Venn diagram analysis comparing our curated list of R-loop regulators with the CRISPRi-identified mitoROS and cellular ROS regulators. As shown in Figure 9A, 20 R-loop regulators overlapped with pro-mitoROS regulators, and 13 overlapped with anti-mitoROS regulators, hereafter referred to as *pro-mitoROS/R-loop regulators* and *anti-mitoROS/R-loop regulators*, respectively.

These findings support the hypothesis that *1)* R-loop regulators modulate mitoROS production (both pro- and anti-mitoROS regulators) through anterograde signaling (nucleus-to-mitochondria communication) (29); *2)* mitoROS regulators can influence R-loop formation via retrograde signaling (mitochondria-to-nucleus feedback) (29); and *3)* pharmacological inhibition of mitoROS such as with mitoTempo (59, 68), may modulate R-loop formation and stability. Pathway enrichment analysis of the 20 pro-mitROS/R-loop regulators revealed significant enrichement in protein-RNA complex organization, mitochondrial organization, RNA catabolic process, regulation of cell cycle phase transition, and DNA templated transcription. In contrast, the 13 anti-mitoROS/R-loop regulators were enriched in pathways related to the Nop56p-associated pre-rRNA complex, SARS-Co-V1 host interaction, and protein maturation. Integration of these datasets with the NRF2^−^/^−^ transcriptomic dataset (GSE71695) revealed that one anti-mitoROS regulator, CYFIP2, is transcriptionally promoted by NRF2.

We also performed Venn diagram analyses comparing R-loop regulators with 283 cellular ROS regulators. As shown in Figure 9B, 14 R-loop regulators overlapped with pro-cellular ROS regulators (13 identified in the main table plus *PBRM1*), and 7 overlapped with anti-cellular ROS regulators. These were thus designated as pro-cellular ROS/R-loop regulators and anti-cellular ROS/R-loop regulators, respectively.

These findings suggest that 1) R-loop regulators may contribute to cellular ROS generation, either by promoting or suppressing ROS production; 2) cellular ROS regulators may influence R-loop formations and genomic stability; 3) pharmacological inhibition of cellular ROS (69, 70) may modulate R-loop dynamics. Notably, *NCOA5*, a pro-cellular ROS regulator, is repressed by NRF2, consistent with its classification as an NRF2-inhibited pro-oxidant gene. Pathway enrichment analysis of the 13 pro-cellular ROS/R-loop regulators revealed significant enrichment in translation, localization within membrane, and negative regulation of intracellular signal transduction.

### 2.9 Dysregulation of mitoROS- and cellular ROS-associated R-loop regulators may contribute to the pathogenesis of metabolic inflammations and influenza infection

To investigate the potential contribution of pro-mitoROS/R-loop regulators and anti-mitoROS/R-loop regulators to disease, we analyzed the expression profiles of 33 known mitoROS/R-loop regulators across multiple disease states, including metabolic inflammation (atherosclerosis, NASH, AAA) and influenza virus-infected ECs. As shown in Table 4, 25 out of 33 mitoROS/R-loop regulators were modulated in these disease conditions. Specifically, two pro-mitoROS/R-loop regulators, *CHEK1* (checkpoint kinase 1) and *HLA-DRB1* (major histocompatibility complex, class II, DR beta 1) were upregulated in atherosclerosis 78 weeks and influenza virus infected ECs, respectively. Two anti-mitoROS/R-loop regulators including *CYFIP2* (cytoplasmic FMR1 interacting protein 2) and *AGPS* (alkylglycerone phosphate synthase) were downregulated in atherosclerosis 32 weeks and NASH respectively; and four anti-mitoROS/R-loop regulators including *SRP72* (signal recognition particle 72), *AGPS*, *TUFM* (tu translation elongation factor, mitochondrial), and *CYFIP2* were downregulated in influenza virus infected ECs. The literature indicated that the 25 mitoROS/R-loop regulators play significant roles in inflammations and diseases (Table 4). These findings suggest that increased expression of pro-oxidant mitoROS regulators and suppressed expression of anti-oxidant mitoROS regulators may lead to enhanced oxidative stress, contributing to R-loop imbalance and promoting chronic inflammation or viral pathogenesis.

**Table 4:** Shared R-loop and mitochondrial ROS regulators implicated in inflammation and disease progression.

Disease conditions	Up/downregulated genes (FC > 1, *P* < 0.05)	33 R-loop regulators overlapped with mitochondrial ROS regulators	Roles of the 33 R-loop regulators shared with mitochondrial ROS regulators in inflammation	PMIDs
AAA 14 days	4 upregulated R-loop regulators	-		
	33 downregulated R-loop regulators	*PRPF4B* (pro-mito ROS regulator)	*PRPF4B* (Pre-mRNA Processing Factor Kinase PRP4K): Significantly upregulated in the nasopharynx during SARS-CoV-2 infection, suggesting a role in the host transcriptional response.	34400346
AAA 28 days	56 upregulated R-loop regulators	-		
	5 downregulated R-loop regulators	-		
Atherosclerosis 32 weeks	39 upregulated R-loop regulators	-		
	12 downregulated R-loop regulators	*CYFIP2* (anti-mito ROS regulator)	*CYFIP2* (Cytoplasmic FMR1 Interacting Protein 2): Deletion of Cyfip2 in postnatal forebrain excitatory neurons leads to enhanced prefrontal neuronal activity and increased social dominance behavior in mice.	33192297
Atherosclerosis 78 weeks	89 upregulated R-loop regulators	*CHEK1* (pro-mito ROS regulator)	*CHEK1* (Checkpoint Kinase 1): Inhibition of CHEK1 combined with low-dose hydroxyurea enhances ATM-mediated, NF-*κ*B-dependent pro-inflammatory chemokine expression in melanoma cells. *CYFIP2* (See earlier description)	40507298 (*CHEK1)*
		*CYFIP2* (anti-mito ROS regulator)		
	25 downregulated R-loop regulators	-		
NASH	19 upregulated R-loop regulators	-		
	3 downregulated R-loop regulators	*AGPS* (anti-mito ROS regulator)	*AGPS* (Alkylglycerone Phosphate Synthase): An ether lipid biosynthetic enzyme that modulates the balance of structural and signaling lipids, promoting cancer progression.	23980144
Monosodium urate (MSU) stimulated LECs	16 upregulated R-loop regulators	-		
	15 downregulated R-loop regulators	-		
Influenza virus-infected endothelial cells.	200 upregulated R-loop regulators	*HLA-DRB1* (pro-mito ROS regulator)	*HLA-DRB1* (Major Histocompatibility Complex, Class II, DR Beta 1): Associated with persistent chronic inflammation, contributing to cardiovascular events and increased mortality in rheumatoid arthritis patients. *PPIG* (Peptidylprolyl Isomerase G): Regulates the spatial and temporal distribution of small ubiquitin-like modifiers during human placental development and in response to oxidative and inflammatory stress.	17266100 (*HLA-DRB1*)
		*PPIG*		29468681 (*PPIG*)
		*METAP2 RPS9* (anti-mito ROS regulator)	*METAP2* (Methionyl Aminopeptidase 2): Involved in angiogenesis and endothelial cell proliferation, representing a validated therapeutic target in cancer treatment. *RPS9* (Ribosomal Protein S9): Its silencing activates p53 and inhibits cancer cell growth through multiple cellular pathways.	38973915 (*METAP2*) 20221446 (*RPS9*)
	551 downregulated R-loop regulators	*XRN2 NDUFS1 MRPL12 CHEK1 KIF5B GRSF1 PRPF4B RDX AGK STRAP PPP1CB NDUFA9 SSBP1* (pro-mito ROS regulator)	*XRN2* (5'-3’ Exoribonuclease 2): Degrades mature let-7 microRNAs in multiple human cancer cell lines, regulating tumor suppressor miRNAs with critical pathophysiological roles. *NDUFS1* (NADH: Ubiquinone Oxidoreductase Core Subunit S1): Mutations cause metabolic reprogramming and impaired electron transport. *MRPL12* (Mitochondrial Ribosomal Protein L12): Promotes hepatocellular carcinoma by regulating mitochondrial biogenesis and metabolism. *CHEK1* (See earlier description). *KIF5B* (Kinesin Family Member 5B): Dysregulated KIF5B and RHEB impair mitophagy, inducing mitochondrial ROS-mediated NLRP3 inflammasome activation and exacerbating lipotoxicity. *GRSF1* (G-Rich RNA Sequence Binding Factor 1): Loss of GRSF1 activates mTOR, triggering proinflammatory transcriptional programs. *PRPF4B* (PRP4K, Pre-mRNA Processing Factor Kinase PRP4K): Cooperates with Foxa1/2 to regulate alternative splicing during thymocyte development. *RDX* (Radixin): Regulates endothelial barrier function following thrombin stimulation. *AGK* (Acylglycerol Kinase): Maintains metabolic homeostasis and immune responses in CD8+ T cells. *STRAP* (Serine/Threonine Kinase Receptor Associated Protein): Key modulator of cancer-associated signaling pathways. *PPP1CB* (Protein Phosphatase 1 Catalytic Subunit Beta): Potential therapeutic target in inflammation-related diseases. *NDUFA9* (NADH: Ubiquinone Oxidoreductase Subunit A9): Stabilizes mitochondrial complex I structure. *SSBP1* (Single-Stranded DNA Binding Protein 1): Biomarker linked to ferroptosis in glioblastoma. *SRP72* (Signal Recognition Particle 72): Mutations linked to familial aplasia and myelodysplasia. *TUFM* (Tu Translation Elongation Factor, Mitochondrial): Regulates mitochondrial protein quality control and programmed cell death; implicated in viral infections and cancer.	31557978 (*NDUFS1*) 38104924 (*MRPL12*) 25462067 (*KIF5B*) 30753671 (*GRSF1*) 34323272 (*PRPF4B*) 23729486 (*RDX*) 31204281 (*AGK*) 40558481 (*STRAP*) 35665742 (*PPP1CB*) 23223238 (*NDUFA9*) 36180956 (*SSBP1*) 22541560 (*SRP72*) 38868764 (*TUFM*)
		*SRP72 AGPS TUFM CYFIP2* (anti-mito ROS regulator)		
			*AGPS* and *CYFIP2* (See earlier descriptions).	

Among the 33 R-loop regulators that overlap with mitochondrial ROS regulators, several demonstrate altered expression patterns associated with specific disease contexts. Notably, pro-mitochondrial ROS regulators such as CHEK1 are upregulated in advanced atherosclerosis (78 weeks), while HLA-DRB1 also shows increased expression. In contrast, downregulation of six anti-mitochondrial ROS regulators—including CYFIP2 in atherosclerosis (32 weeks), AGPS in non-alcoholic steatohepatitis (NASH), and SRP72—is observed. Additionally, AGPS, TUFM, and CYFIP2 are downregulated in the context of influenza virus infection, potentially contributing to disease progression. Collectively, these 33 shared R-loop and mitochondrial ROS regulators are implicated in the regulation of inflammation and the pathogenesis of various diseases.

We also examined the expression patterns of 21 cellular ROS-associated R-loop regulators, with 19 found to be differentially expressed in metabolic inflammatory and viral infection settings (Table 5). One pro-cellular ROS/R-loop regulator, *PTPRC* (CD45) (protein tyrosine phosphatase receptor type C), was upregulated in AAA 28 days, atherosclerosis 32 and 78 weeks, and influenza virus infected ECs. CD45 is a pan-leukocyte marker and immune cell marker (55) with tyrosine phosphatase activity involved in the regulation of signal transduction in hematopoiesis, suggesting enhanced leukocyte infiltration and immune activation in these disease contexts. One anti-cellular ROS/R-loop regulator, *TRDMT1* (TRNA Aspartic Acid Methyltransferase 1) was downregulated in atherosclerosis 78 weeks; and five anti-cellular ROS/R-loop regulators such as PFKP (phosphofructokinase, platelet), *SCYL1* (SCY1 like pseudokinase 1), *ME2* (malic enzyme 2), *SRPK2* (SRSF protein kinase 2), and EXOG (exo/endonuclease G) were also downregulated in influenza virus infected ECs. The literature indicated that the 19 cellular ROS/R-loop regulators play significant roles in inflammations and diseases (Table 5). Downregulation of these anti-ROS regulators likely reduces the cell's capacity to buffer ROS, facilitating oxidative DNA damage, R-loop misregulation, and innate immune reprogramming.

**Table 5:** Differential regulation of R-loop regulators shared with cellular ROS regulators across disease stages.

Disease conditions	Up/downregulated genes (FC >1, *P* < 0.05)	Ratio of upregulated to downregulated R-loop regulators	Expression of 21 R-Loop regulators overlapping with cellular ROS regulators across seven disease models	Roles of the 21 R-loop regulators shared with cellular ROS regulators in inflammation	PMIDs
AAA 14 days	4 upregulated R-loop regulators	0.12 (early AAA)	-		
	33 downregulated R-loop regulators		-		
AAA 28 days	56 upregulated R-loop regulators	11.2 (late AAA)	*PTPRC* (pro-cellular ROS regulator)	*PTPRC* (protein tyrosine phosphatase receptor type C, also known as CD45): A key regulator of innate immune signaling, with emerging roles in regenerative medicine.	34039664
	5 downregulated R-loop regulators		-		39058408
Atherosclerosis 32 weeks	39 upregulated R-loop regulators	3.25 (full-developed atherosclerosis)	*PTPRC* (pro-cellular ROS regulator)	*PTPRC* (See earlier description above).	
	12 downregulated R-loop regulators		-		
Atherosclerosis 78 weeks	89 upregulated R-loop regulators	3.56 (advanced atherosclerosis)	*PTPRC* (pro-cellular ROS regulator)	*PTPRC* (See earlier description above). *CSNK1A1* (casein kinase 1 alpha 1): heterozygous multipotent progenitor cells exhibit a pro-proliferative gene expression, while hematopoietic stem cells show reduced inflammatory signaling and immune response. Also, cisplatin-induced acute kidney injury is associated with upregulation of *CSNK1A1*, suggesting a potential role in renal stress response.	35016204
			*CSNK1A1* (anti-cellular ROS regulator)		35059728
	25 downregulated R-loop regulators		*ARHGAP17* (pro-cellular ROS regulator)	*ARHGAP17* (Rho GTPase Activating Protein 17): Regulates monocyte differentiation and inflammatory responses in coordination with RUNX3. *TRDMT1* (tRNA Aspartic Acid Methyltransferase 1): Protects against LPS-induced inflammation by modulating the TLR4–NF-κB/MAPK–TNF-α signaling axis.	37371794 (ARHGAP17) 35474613 (TRDMT1)
			*TRDMT1* (anti-cellular ROS regulator)		
NASH	19 upregulated R-loop regulators	6.33 (chronic, NASH)	-		
	3 downregulated R-loop regulators		-		
Monosodium urate (MSU) stimulated LECs	16 upregulated R-loop regulators	1.07 (acute stimulation)	-		
	15 downregulated R-loop regulators		-		
Influenza virus-infected endothelial cells.	200 upregulated R-loop regulators	0.36 (acute infection and inflammation)	*PTPRC* (pro-cellular ROS regulator)	*PTPRC, TRDMT1, and CSNK1A1* (See earlier description above)	
			*TRDMT1 CSNK1A1* (anti-cellular ROS regulators)		
	551 downregulated R-loop regulators		*UBA1 FARSB AGK RPS24 DIMT1 ACP1 SUPT6H* (pro-cellular ROS regulators)	UBA1 (Ubiquitin-Like Modifier Activating Enzyme 1): Somatic mutations in UBA1 cause VEXAS syndrome, an autoinflammatory disorder characterized by systemic inflammation and hematologic abnormalities. FARSB: FARSA deficiency is associated with autoimmunity and elevated interferon signaling in all reported patients. AGK (Acylglycerol Kinase): A component of the TIM22 mitochondrial import complex, AGK maintains mitochondrial integrity through a kinase-independent mechanism and interacts with complex I. RPS24 (Ribosomal Protein S24): A core ribosomal protein with a microglia-specific isoform (Rps24c) upregulated during neuroinflammation due to aging or neurodegenerative insults. DIMT1 (DIM1 RRNA Methyltransferase And Ribosome Maturation Factor): Regulates *β*-cell protein synthesis, mitochondrial function, and insulin secretion. ACP1 (Acid Phosphatase 1): The AA genotype may offer protective effects against cardiovascular risk factors. SUPT6H (SPT6 Homolog, Histone Chaperone and Transcription Elongation Factor): Loss of SUPT6H impairs lncRNA regulation, leading to R-loop formation, replication stress, and cellular senescence.	40445522 (UBA1) 35132614 (FARSB) 35547757 (AGK)
					35148993 (DIMT1) 19570551 (ACP1) 30449723 (SUPT6H)
			*PFKP SCYL1 ME2 SRPK2 EXOG* (anti-cellular ROS regulators)	PFKP (Phosphofructokinase, Platelet): A key glycolytic enzyme involved in regulating cell proliferation, apoptosis, autophagy, migration, and stemness through both glycolysis-dependent and -independent pathways. Highly expressed in certain immune cell types. SCYL1 (SCY1 Like Pseudokinase 1): Mutations in SCYL1 are linked to CALFAN syndrome, characterized by low *γ*-glutamyl-transferase cholestasis, acute liver failure, and neurodegeneration. ME2 (Malic Enzyme 2): Supports metabolic fitness and anti-tumor function of CD8⁺ T cells. SRPK2 (SRSF Protein Kinase 2) Regulates splicing via phosphorylation of SRSF proteins; aberrant activity contributes to pathogenic splicing and disease, highlighting its therapeutic relevance. EXOG (Exonuclease G): A mitochondrial nuclease involved in DNA degradation, genome maintenance, inflammation, and cell death responses.	37704285 (PFKP) 29419818 (SCYL1) 39151423 (ME2) 37655328 (SRPK2) 35883600 (EXOG)

This table presents the differential expression patterns of 21 R-loop regulatory genes that are also identified as cellular ROS regulators via CRISPR interference (CRISPRi) screening. The ratio of upregulated to downregulated R-loop regulators increases in the later stages of both AAA and atherosclerosis, suggesting progressive activation of R-loop–associated inflammatory signaling during disease progression. In contrast, this ratio is less than 1 in influenza virus infection, indicating predominant downregulation of these genes. Notably, the pro-oxidant regulator PTPRC (CD45) was consistently upregulated across advanced stages of AAA (28 days), atherosclerosis (32 and 78 weeks), and influenza infection. The anti-oxidant regulator CSNK1A1 showed upregulation in both late-stage atherosclerosis (78 weeks) and influenza infection. In contrast, TRDMT1, another anti-oxidant regulator, exhibited opposing trends: downregulated in late-stage atherosclerosis but upregulated in influenza infection. Collectively, the shared regulation patterns of these R-loop/ROS-associated genes support their involvement in chronic inflammation and oxidative stress, potentially contributing to disease pathogenesis across distinct inflammatory conditions.

Taken together, our data reveals that three pro-oxidant R-loop regulators are upregulated in disease: *CHEK1 and HLA-DRB1* (mitoROS-associated) and *PTPRC (CD45)* (cellular ROS-associated); twelve antioxidant R-loop regulators are downregulated in disease: six mitoROS-associated: *CYFIP2, AGPS, SRP72, TUFM* (including CYFIP2 and AGPS downregulated in multiple conditions) and six cellular ROS-associated: *TRDMT1, PFKP, SCYL1, ME2, SRPK2,* and *EXOG.*

These patterns of increased pro-ROS and decreased anti-ROS R-loop regulators support the notion that ROS dysregulation directly impacts R-loop homeostasis, contributing to persistent inflammation, genomic instability, and disease progression in metabolic disorders and viral infections.

## 3 Discussion

We and others have previously reported that RNA-based regulatory mechanisms such as alternative splicing (71, 72), RNA methylation (73), and non-coding RNAs including microRNAs (25–27, 74–77) and circular RNAs (78, 79)), play essential roles in cell death, the generation of autoantigens/neoantigen epitopes (80–82), and the pathogenesis of various diseases. R-loops, three-stranded nucleic acid structures composed of a DNA:RNA hybrid and the associated non-template single-stranded DNA. R-loops may form under a variety of circumstances and may be tolerated or actively cleared by cellular components; experimentally, they can be generated during transcription of DNA sequences (e.g., high-GC regions) that favor annealing of nascent RNA behind the progressing RNA polymerase, and at least ∼100 bp of DNA:RNA hybrid is required to form a stable R-loop structure. R-loops have recently emerged as important regulators of genomic stability and inflammation. Accumulating evidence suggests that R-loops can activate innate immune responses (83), induce mitochondria-dependent epithelial cell necroptosis, and trigger inflammatory cascades (10, 84). For example, NAT10-dependent acetylation of R-loops restrains their accumulation and may modulate gene expression in a subset of inflammation-related genes (12). However, the abundance of R-loops in genomic regions of genes associated with metabolic inflammatory diseases and infections—such as atherosclerosis, AAA, NASH, gout, and influenza—remains poorly understood. Specifically, it was unknown whether proinflammatory genes upregulated in these conditions are associated with increased or decreased R-loops. The genomic regions refer broadly to the genomic DNA regions encoding proinflammatory genes that can be transcribed into single-stranded pre-mRNA prior to intron removal (https://en.wikipedia.org/wiki/R-loop), rather than a fixed window such as TSS ±2 kb. This framing is consistent with prior observations that intron-containing genes display decreased R-loop numbers and decreased DNA damage compared to intron-less genes of similar expression in both yeast and humans. Importantly, R-loop functions can vary substantially depending on their position along the transcription unit (e.g., promoter/TSS-proximal vs. terminator/3′ regions), and future position-resolved mapping in relevant primary cells and tissues will be required to refine these location-dependent interpretations in the disease contexts analyzed here.

In this study, we provide novel insights through integrative analysis of transcriptomic datasets and R-loop profiling, we made the following novel findings: ***1)*** The number of R-loops in genomic regions encoding the top 49 and 50 upregulated genes at days 14 and 28 after Ang-II infusion in ApoE^−^/^−^ aortas (an AAA mouse model) is significantly decreased compared to those encoding downregulated genes. ***2)*** Similarly, the number of R-loops in genomic regions encoding 50 genes upregulated during atherosclerosis (at 32 and 78 weeks of HFD-feding in ApoE^−^/^−^ aortas) is significantly lower than in downregulated genes. ***3)*** In NASH patients' livers, the top 36 upregulated genes have fewer R-loops than the top 38 downregulated genes. ***4)*** In MSU-stimulated LECs, the top 50 upregulated genes show reduced R-loop numbers compared to the top 50 downregulated genes. ***5)*** In influenza virus-infected HUVECs, the top 50 upregulated genes show reduced R-loop numbers compared to the top 50 downregulated genes. ***6)*** The ratio of R-loops in regions encoding disease-upregulated vs. disease-downregulated genes increases with disease progression, suggesting that reduced R-loop numbers in upregulated genes may result from innate immune reprogramming of R-loop regulatory proteins. A key unanswered question is why proinflammatory loci that are transcriptionally activated across multiple disease contexts would be associated with reduced R-loop signals. One plausible interpretation is that robust transcriptional activation creates a local “transcriptional stress” state that engages transcription-coupled R-loop surveillance and removal, resulting in fewer persistent/steady-state R-loops despite high RNA output. Importantly, the functional consequences of R-loops can be location dependent: promoter/TSS-proximal R-loops have been linked to transcription initiation and chromatin regulation, gene-body R-loops may arise during elongation and co-transcriptional RNA processing, and terminator-associated R-loops can contribute to transcription termination. Therefore, the apparent decrease observed here may reflect a net balance between formation and accelerated resolution that differs across promoter, coding, and terminator regions. Definitive dissection of these mechanisms will require future locus-specific, position-resolved R-loop mapping and temporal/perturbation experiments in relevant primary cells/tissues. An additional mechanistic possibility is that reduced R-loop numbers at proinflammatory loci reflects enhanced R-loop resolution by canonical removal factors, including RNase H enzymes and helicases such as Senataxin (SETX) and Aquarius (AQR). Differential expression of the SETX target gene set has been predicted to activate multiple neurodegenerative pathways (85), and system-level studies have identified the SETX target gene set as showing consistent, significant signals in cellular stress and disease contexts, including fluid shear stress and atherosclerosis (86). Moreover, the AQR/PLAU signaling axis has been implicated in cellular senescence in hyperglycemic conditions and endothelial dysfunction (87). However, at present we did not find clear literature support that over-activation of RNase H1 or specific helicases is the dominant driver of the R-loop reductions observed across the five diseases analyzed here, highlighting the need for future experimental work to directly measure R-loop resolution activity at selected loc. Several genomic and epigenomic features are known to influence R-loop formation and stability, including gene length, GC content, CpG density, G-quadruplex (G4)-forming sequences, chromatin accessibility, transcriptional activity, and RNA-processing dynamics. Because the primary objective of the present study was to identify common associations between disease-associated transcriptional changes and experimentally annotated R-loop abundance across multiple independent datasets, these variables were not incorporated into the current analytical framework. Consequently, although the observed relationships were remarkably consistent across five independent inflammatory disease models, we cannot exclude the possibility that some of the observed differences are partially influenced by these genomic characteristics. Future studies integrating disease-specific R-loop mapping with multivariable regression models, matched-gene analyses, and additional genomic annotations will be important for determining the independent contribution of R-loop abundance to inflammatory gene regulation while controlling for these potential confounding variables. ***7)*** The antioxidant transcription factor NRF2 promotes the expression of 10 R-loop regulatory proteins and suppresses the expression of 17 others. In NRF2-deficient states (e.g., in metabolic diseases or influenza infection), 8 pro-ROS R-loop regulators are upregulated, while 8 anti-ROS regulators are downregulated—potentially contributing to disease progression. ***8)*** Twenty R-loop regulators overlap with CRISPRi-identified pro-mitochondrial ROS (mitoROS) regulators, 13 with anti-mitoROS regulators, 14 with pro-cellular ROS regulators, and 7 with anti-cellular ROS regulators, suggesting extensive mutual regulation between R-loop and ROS pathways. ***9)*** Specifically, the upregulation of pro-mitoROS/R-loop regulators CHEK1 and HLA-DRB1, and the pro-cellular ROS/R-loop regulator PTPRC, along with the downregulation of anti-mitoROS regulators (CYFIP2, AGPS, SRP72, TUFM) and anti-cellular ROS regulators (TRDMT1, PFKP, SCYL1, ME2, SRPK2, EXOG), may play pathogenic roles in metabolic inflammation and influenza virus infection.

Among the R-loop regulatory proteins (RLPs) highlighted across models, G3BP1/2 and DHX9 emerge as translationally relevant candidates. Pharmacological modulation of stress granules via G3BP1/2 has been proposed as a therapeutic pathway in cancer, inflammatory disease, and neurodegeneration (88), suggesting that stress-granule/RNP homeostasis may be a tractable node connecting cellular stress responses to inflammatory transcriptional programs. In addition, a potent and selective small-molecule inhibitor of DHX9 has been reported to abrogate proliferation of microsatellite instable cancers with deficient mismatch repair (89), supporting the feasibility of targeting DHX9 pharmacologically. Notably, DHX9 has also been reported to strengthen atherosclerosis progression by promoting inflammation in macrophages (90), providing a mechanistic link between an RLP identified in our integrative analyses and inflammatory cardiovascular pathology.

**Figure 10:**
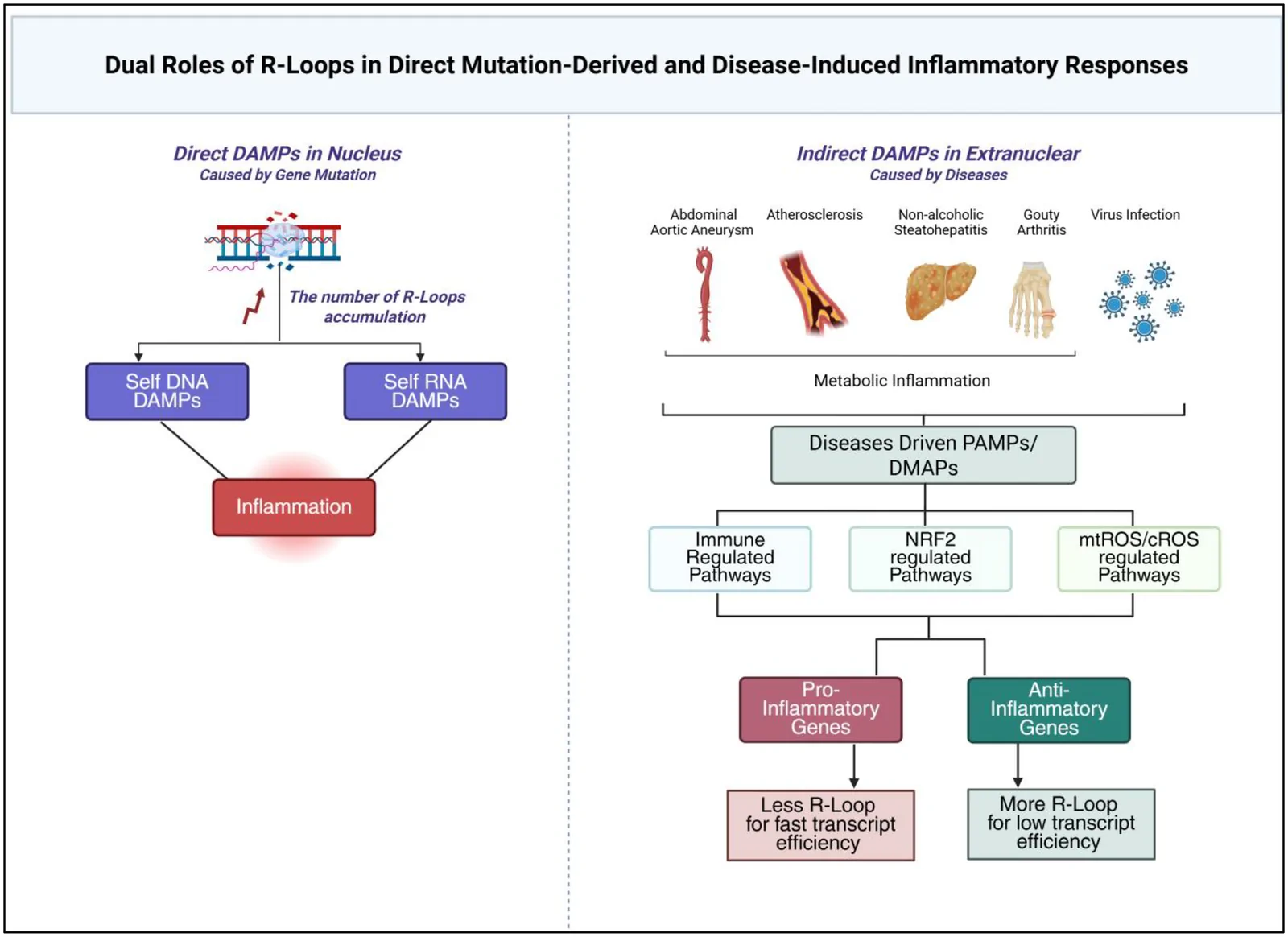
Working model illustrating the distinct roles of R-loops in mutation-induced and disease-driven inflammation and their regulation via the NRF2–ROS–R-loop axis. This schematic summarizes the dual functions of R-loops in inflammatory regulation: Left panel: In the nucleus, DNA or RNA mutations intrinsically generate self-DNA and RNA danger-associated molecular patterns (DAMPs), acting as direct nuclear DAMPs. R-loops amplify these mutation-derived DAMPs, promoting inflammation, consistent with the classical view that excessive R-loops cause genomic instability and trigger inflammatory responses. Right panel: Conversely, in disease-derivn inflammation (e.g., AAA, atherosclerosis, NASH, gouty arthritis, and influenza virus infection), DAMPs and pathogen-associated molecular patterns (PAMPs) activate extranuclear receptors such as TLRs, NLRP3 inflammasome, and the cGAS-STING pathway. These signaling cascades indirectly modulate R-loop dynamics, leading to decreased R-loop numbers at proinflammatory gene loci, thereby facilitating rapid transcriptional activation and robust inflammatory responses. Meanwhile, anti-inflammatory and homeostatic gene loci maintain elevated R-loop numbers, restraining their transcription. Crucially, the NRF2–ROS–R-loop axis serves as a key regulatory network: NRF2 upregulates 10 R-loop regulators and downregulates 17 others. Loss of NRF2 shifts this balance toward pro-ROS R-loop regulators (e.g., CHEK1, HLA-DRB1, PTPRC) while suppressing anti-ROS R-loop regulators (e.g., CYFIP2, AGPS, SRP72, TUFM, TRDMT1, PFKP), thereby exacerbating inflammation. Together, these findings reveal a novel mechanism whereby reduced R-loop numbers enhance proinflammatory gene transcription in disease-associated inflammation and highlight the NRF2–ROS–R-loop network as a promising therapeutic target in metabolic inflammation, cardiovascular diseases, immune disorders, and cancer.

Based on these findings, we propose a new working model (Figure 10): First, R-loop numbers are significantly lower in genomic regions encoding the most upregulated genes across models of AAA (Ang-II infusion in ApoE^−^/^−^ mice), atherosclerosis (HFD-fed ApoE^−^/^−^ mice), NASH (human liver), MSU-stimulated LECs, and influenza-infected ECs, compared to downregulated genes. This suggests that reduced R-loops in proinflammatory gene regions may facilitate their rapid and efficient transcription in response to DAMPs and PAMPs. Conversely, increased R-loop formation in genomic regions of downregulated genes may impede their transcription, contributing to loss of homeostatic gene expression. ***Second***, contrary to the prevailing notion that excessive R-loops promote inflammation via genomic instability, our results indicate that decreased R-loops in proinflammatory gene regions in the genome drive inflammation in response to extranuclear DAMP/PAMP signaling. To reconcile this discrepancy, we propose that excessive R-loops serve as intranuclear DAMPs, triggering inflammation via genomic instability, while in the context of metabolic inflammation and infection, decreased R-loops in key gene loci in the genome may enable higher transcriptional efficiency of inflammatory genes. Excessive R-loops can act as intranuclear DAMPs in two forms self-DNA-derived DAMPs, which activate nuclear stress and inflammation through 10 self-DNA DAMP receptors including absent in melanoma 2 (AIM2), C-type lectin domain family 2 member D (CLEC2D), cyclic GMP-AMP synthase (cGAS), TLR9 (located on membranes of acidified endosomes and endolysosomes) (91), NLRP3, NLRP10, Z-DNA binding protein 1 (ZBP1), advanced glycosylation end-product specific receptor (RAGE), interferon gamma inducible protein 16 (IFI16), coiled-coil domain containing 25 (CCDC25) (92), as well as ATM- and Rad3-related (ATR) and Ataxia-telangiectasia-mutated (ATM) kinases in the activation of an R-loop-dependent DNA damage response (93). In addition, self-RNA-derived DAMPs trigger inflammation and immunity (94) via 10 self-RNA DAMP receptors including interferon induced with helicase C domain 1 (IFIH1, MDA5), ZBP1, RNA sensor retinoic acid-inducible gene 1 (RIG-I), mitogen-activated protein kinase kinase kinase (MAPKKK) ZAKa (95), DEAD (Asp-Glu-Ala-Asp)-Box Helicase 17 (DDX17) (92), three TLRs located on membranes of acidified endosomes and endolysosomes such as TLR3 (94), TLR7, and TLR8 (92), TLR13 and DExH-box helicase 58 (LGP2) (94). ***Third***, In contrast to increased and excessive R-loops triggered by nuclear stresses, it has been reported that atherosclerotic cardiovascular diseases (including atherosclerosis and AAA)-related DAMPs such as oxidized low-density lipoprotein (oxLDL) (16) and cholesterol crystal trigger inflammation via 10 membrane Toll-like receptors (TLRs), lectin-like oxLDL receptor-1 (LOX-1) (96), membrane C-type lectin domain family 4 member E (CLEC4E, Mincle) (97) and cytosolic inflammasomes (16), membrane advanced glycosylation end-product specific receptor (RAGE), cytosolic cGAS-the cyclic GMP–AMP receptor stimulator of interferon genes (STING), and cytosolic NLRP3 inflammasome (91). Ang-II triggers membrane ang-II receptor type 1 (AGTR1, AT1R) (20, 98). NASH DAMP receptors include membrane TLRs, membrane purinergic receptor P2X7 (P2X7) and cytosolic NLRP3 inflammasome (99). Gouty-arthritis DAMP, MSU stimulation triggers cytosolic NLRP3 inflammasome. Influenza virus infection-derived PAMPs triggers membrane sialylated glycan receptors (100) and other 19 PAMPs/DAMPs receptors including TLR3 for double stranded RNA (dsRNA), TLR7 (for single stranded RNA, ssRNA), TLR8 (for ssRNA), TLR9 (for hypomethylated CpG DNA), TLR2 (for virus coat proteins, VCPs), TLR6 (for VCPs), TLR4 (for VCPs), RIG-I (for dsRNA), MDA5 (Melanoma differentiation associated gene 5 for dsRNA), Langerin (for glycosylated proteins, GPs), MR (for GPs), MDL-1 (for myeloid DAP-12- associating lectin for GPs), MGL (macrophage galactose-type lectin for GPs), DC-SIGN (dendritic cell-specific intercellular adhesion molecule-grabbing nonintegrin for GPs), L-SIGN (liver/lymph node-specific ICAM-3 grabbing non-integrin for GPs), NLRPs (for dsRNA and ATP), AIM2 (for dsDNA), IFI16 (for dsDNA), and cGAS (for dsDNA) (101). All of these DAMPs/PAMPs receptors for atherosclerosis model, AAA model, NASH model and MSU stimulation and some pathways of influenza virus infection in this study are localized outside of nucleus. ***Fourth***, to determine whether extranuclear DAMPs receptor signaling pathways upregulate proinflammatory genes and downregulate non-inflammatory supporting genes by modulating R-loop numbers in the genomic regions encoding those proinflammatory and anti-inflammatory/homeostasis genes, by analyzing a comprehensive experimental database R-loopbase, we made the following significant findings: extranuclear DAMPs and PAMP signals in metabolic cardiovascular diseases such as atherosclerosis and AAA-, NASH-, gouty arthritis-, and influenza virus infection-, upregulated proinflammatory genes are encoded in the genomic regions with decreased R-loops. Since the R-loopbase contains the datasets collected in vascular ECs and VSMCs, our findings are patho-physiologically relevant. Of note, future work is warranted to verify our results with additional experiments. One explanation for our findings is that the genomic regions encoding the genes downregulated in acute influenza virus infected HUVECs, MSU-stimulated LECs and chronic metabolic cardiovascular inflammations have higher numbers of R-loops, which may “slow down” gene transcription and gene upregulation and make gene transcription less efficient in response to extranuclear DAMPs and PAMPs stimulations. ***Fifth***, Oxidative stress is an important and pervasive physical stress encountered by all kingdoms of life and RNAs are sensors of oxidative stress (102). A list of TFs including NRF2 (17), hypoxia-inducible factor (HIF)-1a (103), activator protein-1 (AP-1), NF-kB (104), CCAAT/enhancer-binding protein delta (CEBPD), specificity protein-1 (SP-1), Sirtuins (43, 105), FOXO (106), tumor protein P53 and peroxisome proliferator-activated receptor gamma (PPARG) coactivator 1 alpha (PGC-1*α*) (105) regulate ROS generation and/or being regulated by ROS. Thus, we examined a new hypothesis that ROS are a new mechanism and regulate the expressions of R-loop regulators. First, to demonstrate the proof of principle, we found the following results: 1) anti-oxidant transcription factor NRF2 promotes the expressions of 10 R-loop regulators and inhibits the expressions of 17 R-loop regulators; and 2) the upregulation of these 8 NRF2^−^/^−^ upregulated (pro-ROS) R-loop regulators and downregulation of 8 NRF2^−^/^−^ downregulated (anti-ROS) R-loop regulators in metabolic diseases and influenza virus infection may contribute to the pathogenesis of these diseases. We further determined whether recently reported CRISPRi-identified mitochondrial ROS regulators and cellular ROS regulators are partially shared with R-loop regulators. We found that the 20 R-loop regulators are shared with CRISPRi-identified pro-mitochondrial ROS (mitoROS) regulators; the 13 R-loop regulators are shared with anti-mitoROS regulators; the 14 R-loop regulators are shared with pro-cellular ROS regulators and the 7 R-loop regulators are shared with anti-cellular ROS regulators, suggesting that 54 R-loop regulators and ROS regulators are mutually regulated. Finally, we found the upregulation of two pro-mitoROS/R-loop regulators CHEK1 and HLA-DRB1 and one pro-cellular ROS/R-loop regulator PTPRC and the downregulation of six anti-mitoROS/R-loop regulators such as CYFIP2, AGPS, SRP72, AGPS, TUFM, and CYFIP2 and six anti-cellular ROS/R-loop regulator TRDMT1, PFKP, SCYL1, ME2, SRPK2, and EXOG may contribute to the pathogenesis of metabolic inflammations and influenza virus infection. Collectively, our results have provided insights into the roles of decreased R-loop numbers in the genomic regions for potential higher efficiency upregulation of proinflammatory genes in metabolic inflammations and influenza virus infection as well as pathological functions of NRF2 regulating ROS-sensitive R-loop regulators and ROS/R-loop dual regulators.

Finally, the mechanistic hypotheses proposed in this study warrant direct experimental investigation. Representative inflammatory genes identified in the present analyses, such as IL6, TNF, CCL2, and CXCL10, could be examined by locus-specific DRIP-qPCR or related approaches in disease-relevant cellular models to validate changes in R-loop abundance. Likewise, perturbation of R-loop regulatory pathways through RNase H1 overexpression, catalytically inactive RNase H1 controls, modulation of helicases such as SETX, or pharmacological and genetic manipulation of NRF2/ROS signaling may provide important mechanistic insight into the causal relationship among oxidative stress, R-loop homeostasis, and inflammatory gene transcription. Such studies represent important future directions arising from the present work.

## 4 Materials and methods

### 4.1 Public transcriptomic dataset acquisition and differential expression screening

Transcriptomic datasets were retrieved from the publicly curated repository NCBI Gene Expression Omnibus (https://www.ncbi.nlm.nih.gov/gds), following data processing principles previously established by our group (77, 107, 108). The datasets analyzed in this study include: (1) bulk aortic RNA sequencing profilling from an experimental AAA induced by angiotensin II infusion delivered via subcutaneous osmotic pump in ApoE-/- mice (14- and 28-day infusion vs. saline controls, *n* = 5 per group; GSE17901) (21); (2) aortic microarray profilling from a long term diet -induced atherosclerosis model comparing ApoE-/- mice and wild-type controls maintained on HFD for 32 and 78 weeks (*n* = 3 per group; GSE10000) (109); (3) microarray profilling of human liver samples from clinically diagnosed NASH subjects (*n* = 9) and matched non-NASH controls (*n* = 7; GSE63067); (4) lymphatic endothelial transcriptome from murine LECs treated with 300 μg/ml MSU for 24 h vs. PBS-treated controls (*n* = 3; GSE199950); (5) microarray profiling of human HUVECs following infection with influenza A virus (*n* = 2; GSE59226); and (6) transcriptomic profiling of BMDMs isolated from Nrf2-wild-type and Nrf2-knockout mice (*n* = 4; GSE71695) (Figures 2A,B). These datasets collectively provide genome-wide expression profiles generated from human or murine tissues and immune-responsive cell populations experimentally or clinically exposed to inflammatory, fibrotic, metabolic, or viral-induced immune stress conditions.

Initial differential expression screening was performed using the standardized analysis interface of GEO2R. Genes were considered differentially expressed when meeting the predefined criteria of: (i) nominal *p* < 0.05, and (ii) absolute fold-change ≥2.0 for upregulation or ≤ –2.0 for downregulation (equivalent to |log2FC| ≥ 1.0). These thresholds were selected to capture strong transcriptional modulation at canonical inflammatory effector loci, including cytokines and chemokines central to inflammatory disease biology, while minimizing low-amplitude variation inherent to heterogeneous multi-tissue resources.

### 4.2 DEG curation, structural organization, and post-filtering in spreadsheet environment

Complete differential expression outputs from GEO2R—including fold-change directionality and associated nominal significance values—were imported into the structured data environment of Microsoft Excel. Post-processing was performed using custom-designed Excel macros previously developed and benchmarked by our group to ensure consistent formatting and filtering. Briefly, macros were applied sequentially to: (1) standardize and annotate gene identifiers derived from GEO2R output, (2) enforce filtering based on reported nominal *p*-values, and (3) apply directional fold-change thresholds (≥2.0 or ≤ –2.0). This step did not involve new inferential statistics, but rather ensured structured organization, reproducibility, and threshold enforcement for downstream integrative analyses.

### 4.3 R-loop locus annotation retrieval and gene-locus intersection strategy

R-loop-associated genomic annotations were obtained from the comprehensive multi-cell reference repository, the RLoopBase (https://rloopbase.nju.edu.cn/) (19, 22). This resource aggregates experimentally validated R-loop and RNA-DNA hybrid regions mapped from multiple normal cell and tissue types using DNA-RNA immunoprecipitation sequencing (DRIP-seq), DRIPc-seq, ssDRIP-Seq, and other related assays (Table 1). Because these annotations reflect intrinsic, locus-driven regulatory properties of well-established innate immune genes and cytokine/chemokine promoters, they were compared to our DEG lists to examine directional concordance between disease-induced expression shifts and pre-validated R-loop numbers at the same gene locus, rather than assuming disease-sample origin matching. Intersected gene lists were generated by overlapping curated DEGs and annotated R-loop-associated loci, to identify inflammation-relevant gene-centric regulatory relationships. The present study utilized the experimentally curated R-loop annotations provided by RLoopBase. No additional peak calling, transcript selection, or peak filtering aggregation was performed. Accordingly, the r-loop numbers analyzed in this study represent experimentally annotated R-loop signals associated with the corresponding genomic loci contained within the RLoopBase resource.

### 4.4 Functional pathway and gene-network enrichment analyses

To determine biological function and disease relevance of curated DEG loci, pathway enrichment was performed using orthogonal annotation platforms. Curated gene lists were analyzed using the commercial biostatistical and network-analysis framework of Ingenuity Pathway Analysis (IPA; Redwood City, CA, USA) to identify significantly enriched canonical pathways, upstream transcriptional regulators, and molecular interaction networks associated with inflammatory and chromatin-structural processes. Complementary functional annotation was conducted using the integrative enrichment portal Metascape (https://metascape.org), applying default parameters to explore additional pathway and ontology support (GO, KEGG, Reactome, and others). This step served as a confirmatory enrichment analysis, not a replacement for the primary reported statistics delivered by GEO2R.

### 4.5 Statistical analysis

All statistical analyses performed beyond the initial DEG output were performed using GraphPad Prism v10.1.2 (GraphPad Software, San Diego, CA, USA). Comparisons between two groups were conducted using two-tailed unpaired Student's *t*-tests. For analysis involving more than one independent variable or multiple groups, two-way ANOVA was performed, followed by Bonferroni-adjusted significance correction. Data are presented as mean ± standard error of the mean (SEM), and statistically significance across all comparisons was defined as adjusted *p*-value ≤ 0.05. Because the objective of this study was to identify global associations between disease-associated gene expression and experimentally annotated R-loop numbers across multiple independent datasets, multivariable regression analyses controlling for genomic features (e.g., gene length, GC content, CpG density, and G-quadruplex abundance) were not performed and remain an important direction for future investigation.

## 5 Conclusions

In summary, our study reveals a previously unrecognized connection between RNA–DNA hybrid dynamics, redox regulation, and inflammatory pathogenesis in metabolic diseases and influenza virus infection. Across multiple inflammation-driven mouse transcriptomes—including the Ang-II–induced AAA, the atherosclerotic progression, the steato-inflammatory liver disease model NASH, and influenza infection—we observe a consistent trend where genes induced by inflammatory stress exhibit reduced R-loop numbers at their regulatory loci, relative to downregulated genes. This decrease in R-loops appears to be associated with disease-induced reprogramming of R-loop regulators under oxidative stress conditions. Furthermore, the redox-sensitive antioxidant transcription factor NRF2 transcriptional axis emerges as a major upstream modulator of numerous R-loop regulators, supporting our hypothesis that disruption of NRF2-mediated redox homeostasis may aggravate inflammation through altered R-loop regulatory dynamics. Moreover, intersections between R-loop regulators and both mitochondrial ROS-control gene sets and cellular oxidative stress networks support their integrated function in maintaining genomic and metabolic equilibrium.

Our correlations assess directional concordance at disease-dysregulated inflammatory gene loci with independently pre-validated R-loop annotations, rather than assuming that R-loop measurements originated from matched disease tissues. The reference resource used for locus-level R-loop directionality, the curated multi-cell genomic annotation repository RLoopBase, compiles experimentally validated RNA–DNA hybrid/R-loop intervals derived from multiple normal cell types and tissues using DRIP-based sequencing and RNA-linked peak-calling methods. Because these data represent intrinsic, durable regulatory features of specific gene loci, this database is suitable for gene-centric, locus-direction correlation analyses, even when not generated from disease-matched equivalents. Collectively, this work provides new mechanistic insight into the interplay between cytokine/chemokine genomic loci, redox signaling, and RNA–DNA structural regulators, and highlights R-loop regulatory nodes as multi-disease therapeutic candidates for metabolic inflammatory pathology and viral-triggered immune inflammation.

## 6 Limitations

Several limitations of this study should be acknowledged. First, this work is based entirely on integrated analysis of publicly available, experimentally generated transcriptomic datasets and a public reference R-loop database, and thus the findings should be interpreted as hypothesis-generating rather than definitive mechanistic proof. Although this approach can reveal biologically meaningful and potentially original concepts from high-throughput experimental data, direct experimental validation in the relevant disease models will be necessary in future studies. Second, R-loops are highly dynamic and cell-type/context dependent. Because RLoopBase compiles data from multiple cell lines, predominantly cancer and stem cell models, the baseline R-loop landscapes represented in this resource may not fully reflect those present in primary vascular, hepatic, or other disease-relevant cells, which can differ in transcriptional programs, chromatin organization, proliferation or differentiation status, and oxidative stress conditions. Third, in the present study, the analyzed genomic regions were interpreted broadly as transcribed gene units rather than as precisely resolved promoter-, gene body-, or terminator-specific R-loop regions. Since the biological effects of R-loops may differ depending on their genomic location, the current analysis does not resolve position-specific R-loop dynamics at individual loci. Finally, because the available datasets are largely cross-sectional, the observed association between decreased R-loop abundance and increased proinflammatory gene expression cannot establish directionality or causality. It therefore remains unclear whether reduced R-loops contribute directly to transcriptional activation or instead reflect a consequence of high transcriptional activity together with enhanced R-loop surveillance and clearance. Future locus-specific, position-resolved, and temporally controlled studies in disease-relevant systems will be needed to clarify these mechanisms.

In addition, the present analyses were designed to identify global transcriptomic associations rather than to construct predictive statistical models of R-loop abundance. Therefore, potential confounding variables known to influence R-loop formation—including gene length, GC content, CpG density, G-quadruplex density, chromatin organization, and basal transcriptional activity—were not explicitly modeled in the present analyses. Incorporating these genomic features into multivariable statistical models, together with effect size estimation and confidence interval reporting, will further strengthen future studies aimed at dissecting the independent determinants of R-loop remodeling in inflammatory diseases.

## Data Availability

The datasets presented in this study can be found in online repositories. The names of the repository/repositories and accession number(s) can be found below: NCBI Gene Expression Omnibus (https://www.ncbi.nlm.nih.gov/gds) (GSE17901, GSE10000, GSE63067, GSE199950, GSE59226, GSE71695).
